# Comparative Physiological and Proteomic Analyses Reveal the Mechanisms of Brassinolide-Mediated Tolerance to Calcium Nitrate Stress in Tomato

**DOI:** 10.3389/fpls.2021.724288

**Published:** 2021-11-17

**Authors:** Yi Zhang, Haoting Chen, Shuo Li, Yang Li, Mukesh Kumar Kanwar, Bin Li, Longqiang Bai, Jin Xu, Yu Shi

**Affiliations:** ^1^College of Horticulture, Shanxi Agricultural University, Jinzhong, China; ^2^Institute of Vegetables and Flowers, Chinese Academy of Agricultural Sciences, Beijing, China; ^3^College of Agriculture and Biotechnology, Zhejiang University, Hangzhou, China

**Keywords:** calcium nitrate, brassinolide, stress responses, antioxidative defence, energy metabolism

## Abstract

Secondary salinization caused by the overaccumulation of calcium nitrate [Ca(NO_3_)_2_] in soils due to excessive fertilization has become one of the major handicaps of protected vegetable production. Brassinolide, a bioactive plant steroid hormone, plays an important role in improving abiotic stress tolerance in plants. However, whether and how brassinolide (BR) can alleviate Ca(NO_3_)_2_ stress remains elusive. Here, we investigated the effects of exogenous BR on hydroponically grown tomato (*Solanum lycopersicum* L.) plants under Ca(NO_3_)_2_ stress through proteomics combined with physiological studies. Proteomics analysis revealed that Ca(NO_3_)_2_ stress affected the accumulation of proteins involved in photosynthesis, stress responses, and antioxidant defense, however, exogenous BR increased the accumulation of proteins involved in chlorophyll metabolism and altered the osmotic stress responses in tomatoes under Ca(NO_3_)_2_ stress. Further physiological studies supported the results of proteomics and showed that the exogenous BR-induced alleviation of Ca(NO_3_)_2_ stress was associated with the improvement of photosynthetic efficiency, levels of soluble sugars and proteins, chlorophyll contents, and antioxidant enzyme activities, leading to the reduction in the levels of reactive oxygen species and membrane lipid peroxidation, and promotion of the recovery of photosynthetic performance, energy metabolism, and plant growth under Ca(NO_3_)_2_ stress. These results show the importance of applying BR in protected agriculture as a means for the effective management of secondary salinization.

## Introduction

The development of protected agriculture has made crop production possible beyond the seasonal barriers (Henry, 2019). However, with the fast-growing development of protected agriculture, secondary salinization in the continuous cropping soil has become increasingly common, which is attributed to excessive fertilization and intensive farming. Secondary salinization has a drastic adverse effect on crop production in protected agriculture (Zhu et al., 2021). As it is different from open-field cultivation, the amount of nitrogen fertilizer used in greenhouse vegetable farming is large, with the main anion being nitrate (NO_3_^–^) and the cation being calcium cation (Ca^2+^) in the soils (Shu et al., 2016; Zhu et al., 2021). Calcium nitrate accumulation is one of the main reasons for secondary salinization in greenhouse soils (Niu et al., 2019).

Calcium nitrate stress not only affects photosynthesis and respiration but also causes severe oxidative damage and metabolic disorder, due to the overproduction of reactive oxygen species (ROS), including oxygen (O_2_^⋅^^–^), hydroxide (OH^⋅^), and hydrogen peroxide (H_2_O_2_; Zhang et al., 2008; Li et al., 2015; Shu et al., 2016; Soares et al., 2016; Fan et al., 2017; Zhen et al., 2018). Induced lipid peroxidation, increased electrolyte leakage, and disrupted protein functions are some common consequences of excessive ROS accumulation. However, in response to salt stress such as secondary salinization, plants recruit an antioxidant defense system comprising of superoxide dismutase (SOD), peroxidase (POD), catalase (CAT), glutathione reductase, and ascorbate peroxidase (APX) to scavenge different types of ROS. In addition, the genes encoding glutathione *S*-transferase and glutathione POD are up-regulated to protect plants from salinization (Poonam et al., 2015).

Proteomics can not only describe the complete proteome of an organism but also compare and identify specific proteins affected by different physiological conditions. Under salinity stress, obvious lignification occurs in the roots of tomato plants, which is closely related to the preferential increase of S-adenosyl methionine. Calcium nitrate stress increases the accumulation of lignin biosynthesis-related proteins in cucumber roots followed by protein modification and the accumulation of degradation-associated proteins, while it decreases the accumulation of sugar metabolism-associated proteins (An et al., 2016). Exogenous spermidine can augment the accumulation of glycolysis-related proteins and fructose-6-phosphate biosynthesis by the action of fructokinase in calcium nitrate [Ca(NO_3_)_2_-] treated cucumber plants, thereby modulating carbohydrate and energy metabolism in plants (Du et al., 2018).

Brassinolide is a multifunctional steroid phytohormone that modulates plant growth and stress responses (Bishop and Yokota, 2001; Wang et al., 2019; Chi et al., 2020; Yan et al., 2020). Brassinolide induces cell elongation by activating the plasma membrane (PM) H^+^-ATPase, as described in the “acid-growth theory” (Anzu et al., 2019). Brassinolide stimulates gibberellin (GA) accumulation by modulating the expression of GA biosynthesis-related genes, thus presenting a synergy with GA in cell elongation (Li and He, 2013; Li et al., 2013; Tong et al., 2014). Genetic and biochemical studies have revealed that brassinolide (BR) binds to the extracellular domain of a receptor kinase (BRI1) to initiate the phosphorylation/dephosphorylation cascade, subsequently modulating gene expression (Yang and Komatsu, 2004; Tang et al., 2008).

Several studies showed that exogenous BR enhances photosynthetic performance, ion homeostasis, and the antioxidant system, resulting in improved plant growth, and development under salt stress (El-Mashad and Mohamed, 2012; Yuan et al., 2014; Arif et al., 2017; Ahmad et al., 2018; Ahammed et al., 2020; Su et al., 2020). An increased BR level due to the enhanced expression of the *DET-2* gene in *Arabidopsis* is positively correlated to increased CAT and SOD activities under low temperatures (Tanveer et al., 2019). Importantly, exogenous BR influences protein biosynthesis as well as the degradation of damaged proteins. For instance, BR alters the accumulation of heat shock proteins, proteases, and antioxidant enzymes in plants (Li et al., 2013; An et al., 2016; Li et al., 2018). However, the effect of BR and Ca(NO_3_)_2_ on plant proteome and the potential mechanism of stress mitigation remain elusive.

In this study, using a set of physiological and proteomics analyses, we investigated the role of exogenous BR in modulating the growth, photosynthesis, and antioxidant defense of hydroponically grown tomato seedlings under Ca(NO_3_)_2_ stress. Our findings reveal crucial mechanisms involved in the BR-enhanced tolerance to Ca(NO_3_)_2_ stress, which could be useful to the development of effective strategies for the management of secondary salinization in protected agriculture.

## Materials and Methods

### Plant Materials and Treatments

Tomato (*Solanum lycopersicum* L. cultivar “Money Maker”) seeds were soaked in water at 55°C for 25 min and then sown in a tray containing a vermiculite matrix. The seedlings with three leaves were transferred to a 1/2-strength Yamasaki tomato nutrient solution (Li C. et al., 2016) for 7 days. Subsequently, the seedlings were transferred to fresh 1/2-strength Yamasaki tomato nutrient solution (Stanleygroup, Shandong, China) without (control) or with (i) foliar spraying with 24-epibrassinolide (BR, 0.1 μmol/L; 24-Epicastasterone, Yuanye Biotechnology Co., Shanghai, China), (ii) root exposure to Ca(NO_3_)_2_ solution (Ca, 100 mmol/L), (iii) foliar spraying with 0.1 μmol/L BR plus root exposure to 100 mmol/L Ca(NO_3_)_2_ solution (Ca+BR) for continued cultivation for 5 days. The concentration used for spraying BR (0.1 μmol/L) was selected based on previous studies (Nie et al., 2019; Li et al., 2020). The entire foliar portion was sprayed with 0.1 μmol/L BR or distilled water once every 2 days, and salt stress treatment was carried out on the day of the second spray. The osmotic potential of 100 mmol/L Ca(NO_3_)_2_ was –0.97 MPa at 25°C, which was determined using a WESCOR Vapro 5600 osmometer (WESCOR, ELITechGroup, Inc, Stoneham, MA, United States). Leaf samples were taken from three randomly selected tomato seedlings with uniform growth for each treatment at different time points. Briefly, all leaflets from the second and third fully expanded leaves of the selected tomato seedlings were collected 1, 3, and 5 days after the salt stress treatment, and mixed abundantly for physiological analysis and protein extraction. The harvested leaf samples were immediately frozen in liquid nitrogen before storing in ultra-low temperature refrigerators to determine the H_2_O_2_, malondialdehyde (MDA), ascorbic acid (AsA), SOD, POD, CAT, and other indicators. The plant material taken 5 days after the salt stress treatment was divided into aboveground and underground parts. They were put in the oven at 105°C for 15 min and kept at 60°C until a constant weight was attained. The second leaf from the top was dried for the amino acid analysis.

### Measurements of Biochemical and Physiological Parameters

The fresh weight (FW) of the leaves was measured by a rapid weighing method as described by Zhang et al. (2018). The chlorophylls of the second fully expanded leaves were extracted with acetone (80%) and the chlorophyll content was determined by monitoring the absorbance at 645 and 663 nm (Ahmad et al., 2018). The protein content and proline content were determined as described by Bradford (1976) and Bates et al. (1973), respectively. Three samples were used for the determination of the chlorophyll and proline content. Photosynthetic measurements were performed on fully expanded leaves (the second leaf from the top) on 5 days of salinity treatment at 10:00–11:00 a.m. A standard leaf chamber (2 × 3 cm^2^) fitted on a portable photosynthesis system (6400XT, Li-Cor Inc., Lincoln, NY, United States) was used at ambient relative humidity: 50–60%; carbon dioxide (CO_2_); 400 μmol^⋅^mol^–1^; flow rate: 500 μmol s^–1^; vapor pressure deficit < 2, and photosynthetically active radiation: 800 μmol^⋅^m^–2^^⋅^s^–1^. Each leaf was allowed sufficient time for equilibration in the chamber until constant readings were obtained. The photosynthetic parameters such as the net photosynthetic rate (Pn), stomatal conductance (Gs), intercellular CO_2_ concentration (Ci), transpiration rate (Tr), the instantaneous water use efficiency (WUE), calculated through Pn/Tr, and the stomatal limitation (Ls) defined as 1-Ci/Ca (Ca denotes the atmospheric CO_2_ concentration) were measured according to Hao et al. (2019). The microstructure of the leaf was prepared by the paraffin sectioning method as described by Li et al. (2020). The leaf sections were observed and photographed under an optical microscope.

Amino acid extraction and determination were carried out as described by Ohtsuki et al. (2016). Approximately 0.1 *g* of the oven-dried sample was soaked in 2% 5-sulfosalicylic acid (1.5 ml) for 24 h. After the centrifugation at 13,681 × *g* for 10 min, the supernatant (800 μl) was diluted to 5 mL with 0.02 mmol/L hydrochloric acid (HCl) and then the diluted supernatant was filtered through a 0.45 μm filter membrane (Linghanglab, Tianjin, China) and placed in a 1.5 ml injection bottle (Linghanglab, Tianjin, China). An amino acid analyzer (L-8800, HITACHI, Chiyoda City, Tokyo, Japan) was used to determine the content of different amino acids. Each treatment was determined with three biological replicates and three technical replications.

The H_2_O_2_ and MDA levels were measured as described by Ahmad et al. (2018). Briefly, the fresh leaf tissue (0.3 *g*) was homogenized with 0.1% trichloroacetic acid (TCA) and centrifuged at 12,000 × *g* for 10 min. Approximately, an equal volume of supernatant, 100 mM potassium phosphate buffer (pH 7.0), and 1M potassium iodide were mixed and the absorbance was noted at 390 nm with a spectrophotometer (Shimadzu UV-2450, Japan). The H_2_O_2_ content was expressed as nmol^⋅^g^–1^ FW. The MDA content was measured on the basis of thiobarbituric acid reaction (Bailly et al., 1996). In brief, a 0.5 *g* sample was macerated in 8 ml of 0.1% (w/v) TCA and centrifuged at 4,830 × *g* for 10 min at 4°C. The supernatants were obtained and mixed with 0.5% (w/v) of TCA made in 5% (w/v) TCA. The reaction mixture was heated at 100°C on a water bath for 20 min; afterward, the mixture was put on ice to stop the reaction. After cooling, a step of centrifugation at 7,888 × *g* for 10 min was done and the absorbance was taken at 450, 532, and 600 nm.

Enzyme extraction has been improved with reference to the Gong et al. (2005) method. One gram of leaf sample was ground into a homogenate, using 8 ml of a pre-cooled sodium phosphate buffer (0.05M, pH7.0). The homogenate was transferred into a 15 ml tube and centrifuged at 4°C 9,661 × *g* for 20 min. The supernatant obtained was used for the determination of the activity of enzymes, such as SOD (EC 1.15.1.1), POD (EC 1.11.1.7), and CAT (EC 1.11.1.6). The SOD activity was analyzed using nitroblue tetrazolium (NBT) as described by Gong et al. (2005). The SOD activity was expressed as units (U) of SOD mg^–1^ of protein, in which one SOD unit is defined as the amount of enzyme that inhibits 50% of the reduction rate of NBT. The determinations of the POD and CAT activity were according to the method of Yang with slight modifications (Yang et al., 2010). The POD and CAT activities were expressed as 1U with a change of 1 per minute in OD_470_ and OD_240_, respectively, and expressed as U mg^–1^ protein. The non-enzymatic antioxidant AsA contents were measured using a V_C_ (vitamin C, ascorbic acid) Assay Kit (A009, Jiancheng, Nanjing, China) by noting the absorbance at 536 nm (Quan et al., 2015).

### Protein Extraction, Digestion, Isobaric Tags for Relative and Absolute Quantification Labeling, and Strong Cation Exchange Chromatography

The total protein was extracted from the fully expanded second and third leaves of the tomato seedlings from top to bottom. Briefly, 1 *g* of the leaf sample from every biological replicate were finely crushed in liquid nitrogen and mixed with 10% (w/v) TCA/acetone solution having 65 mM dithiothreitol (DTT) for 1 h (−20°C). Afterward, the extracted sample was centrifuged for 45 min at 10,000 × *g* and the obtained pellet was vacuum-dried and solubilized in 1/10 volume of SDT buffer (4% sodium dodecyl sulfate (SDS), 100 mM dithiothreitol (DTT), and 150 mM Tris–HCl, pH 8). After being incubated for 3 min, the suspended solution was ultrasonicated (80 w, 10 s ultrasonication at a time, every 15 s, and 10 times), and re-incubated at 100°C for 3 min followed by a step of centrifugation at 13,000 × *g* at 25°C for 10 min. The protein content in each sample was calculated using a Bicinchoninic acid Protein Assay Reagent (Promega, Madison, WI, United States) and the samples were stored at −80°C until use. Protein digestion was performed according to the FASP procedure described by Wiśniewski et al. (2009) and the resulting peptide mixture was marked according to the instructions of the manufacturer (AB SCIEX, Framingham, MA, United States) with 8-plex isobaric tags for relative and absolute quantification (iTRAQ). The detailed description related to the sample preparation, digestion, and analysis is provided in the Supplementary Methods.

### Liquid Chromatography-Electrospray Ionization Tandem Mass Spectrometry (MS/MS)

The LC-MS/MS experiment was performed on a Q Exactive Mass Spectrometer coupled to an Easy nLC (Proxeon Biosystems, now Thermo Fisher Scientific Waltham, MA, United States). A volume of 10 μl of each fraction was injected for the LC-MS/MS analysis. The instrument was run with peptide recognition mode enabled and the detailed information related to the sample preparation and instrument conditioning is provided in the Supplementary Methods. *The LC-MS/MS analysis was carried out at HooGen Biotech, Shanghai, China.* The MS/MS spectra were searched using the Mascot search engine (Matrix Science, London, United Kingdom; version 2.2) embedded in Proteome Discoverer 1.3 (Thermo Electron, San Jose, CA, United States) against the UniProt *S. lycopersicum* database (35,921 sequences, download at 20180118) and the decoy database. The search parameters are provided in the Supplementary Methods. Differentially modulated proteins were identified as proteins with fold change (FC) ratio > 1.20 or <0.83 (*P* < 0.05; Li G. et al., 2017; Zhong et al., 2017).

### Bioinformatic Analysis

The Gene Ontology (GO) program Blast2GO^1^ was adopted to annotate differential expression proteins (DEPs) to create histograms of the GO annotation based on their role in the biological process, molecular function, and cell components. For the DEPs pathway analysis, the Kyoto Encyclopedia of Genes and Genomes (KEGG) database, using the KEGG automatic annotation server (KAAS) program^2^ was used. The GO enrichment analysis of each module was performed using Cytoscape v3.7.2.

### Parallel Reaction Monitoring Analysis

Additionally, the protein accumulation determined by the iTRAQ analysis was further quantified and analyzed through LC-Parallel reaction monitoring (PRM) MS. Complete information related to the LC_PRM/MS analysis is appended in the Supplementary Methods. The analysis of the raw data was carried out *via* the Skyline 3.5.0 software (MacCoss Lab, University of Washington, United States; Peterson et al., 2012), where the intensity of every signal given specific peptide sequence can be measured for each sample after the normalization of each protein with the reference standard.

### Statistical Analysis

All physiological data were checked for statistical significance using ANOVA and presented as the mean ± SD of three biological replicates. Duncan’s multiple range test was applied to compare the means at the *P* < 0.05 level in SPSS (version-21.0). The proteomic experiments were also repeated with three independent biological replicates. The 95% confidence (*Z* score = 1.96) was set to pick the proteins whose distribution was removed from the main distribution. The cut-off values for the up-regulated or down-regulated proteins were taken as 1.2- or 0.83-fold, respectively (Li M. et al., 2017; Zhong et al., 2017).

## Results

### Brassinolide Alleviated Ca(NO_3_)_2_ Stress-Induced Growth Inhibition in Tomato Seedlings

**FIGURE 1:**
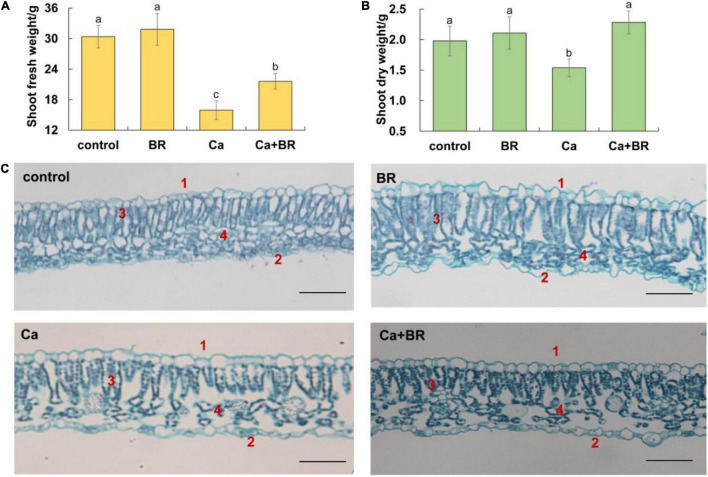
Brassinolide alleviated calcium nitrate [Ca(NO_3_)_2_]-induced growth inhibition in tomato seedlings. **(A)** Shoot fresh weight. **(B)** Shoot dry weight. **(C)** Effects of brassinolide (BR) on the leaf microstructure in Ca(NO_3_)_2_-treated tomato seedlings. 1, Upper epidermis; 2, Lower epidermis; 3, Palisade mesophyll; and 4, Spongy mesophyll. BR, 0.1 μmol/L BR; calcium (Ca), 100 mmol/L Ca(NO_3_)_2_; Ca+BR, 100 mmol/L Ca(NO_3_)_2_ stress plus foliar spraying with exogenous BR. Each data point represents the mean of three independent biological replicates (mean ± SD). Different letters above the bars indicate statistically significant differences (*P* < 0.05). Scale bars = 100 μm.

To elucidate the effects of Ca(NO_3_)_2_-induced salt stress, the growth of tomato seedlings in response to Ca(NO_3_)_2_ treatment was investigated. As shown in Figure 1, Ca(NO_3_)_2_ stress repressed shoot biomass. However, the foliar application of BR alleviated the Ca(NO_3_)_2_-induced shoot growth inhibition in tomato seedlings (Figures 1A,B).

Observations of the microstructure of the tomato leaves showed that Ca(NO_3_)_2_ stress disrupted the arrangement of the epidermis, palisade mesophyll, and spongy mesophyll in tomato leaves (Figure 1C). The intercellular space enlarged, the palisade mesophyll and spongy mesophyll deformed, and the boundaries became blurred after Ca(NO_3_)_2_ stress. Foliar spraying with BR also affected the mesophyll structure in the tomato leaves. Compared with the control, the palisade mesophyll elongated, and the spongy mesophyll arranged chaotically, while the boundaries between the spongy mesophyll, the palisade mesophyll, and the epidermal cells became clear and neatly arranged in the BR-treated tomato leaves. Compared with Ca(NO_3_)_2_ stress alone, the thickness of the upper epidermal cells increased, and the cells were tightly arranged in the Ca+BR treatment. Moreover, the palisade mesophyll was arranged regularly, and an increased number of spongy mesophyll cells with clear cell boundaries were observed in the leaves of Ca+BR-treated tomato seedlings.

### Identification of Differentially Changed Proteins

**FIGURE 2:**
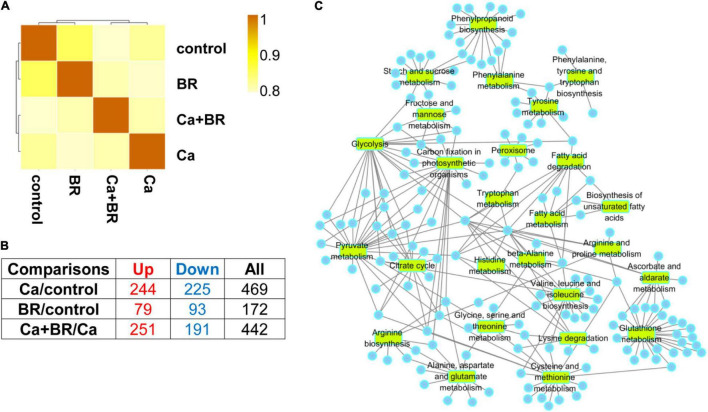
The Spearman correlation coefficient analysis for proteomics data **(A)** and the number of the differentially changed proteins from the leaves of tomato seedlings subjected to 100 mmol/L of Ca(NO_3_)_2_ (Ca), foliar spraying with 0.1 μmol/L BR, or their combinations **(B)**. Up, up-regulated differentially changed proteins. Down, down-regulated differentially changed proteins. **(C)** Selected broad Gene Ontology (GO) terms in the leaves of tomato seedlings subjected to Ca(NO_3_)_2_ stress (Ca), foliar spraying with BR, or their combinations. Circles represent the differentially changed proteins in different pathways.

To elucidate the molecular mechanisms underlying BR-alleviated Ca(NO_3_)_2_ stress, we performed proteomics analysis and revealed the differentially changed proteins (DCPs) using the iTRAQ technique (Figure 2A). A total number of 419,939 secondary mass spectrums were obtained in the Ca(NO_3_)_2_ stress and/or BR-treated seedlings. Among these spectra, 117,757 spectra were matched to the 25,486 identified peptides. Finally, a total number of 25,486 unique peptides and 5,670 proteins were determined [*P* < 0.05, FC > 1.2] (Supplementary Figure 1A and Supplementary Tables 1, 2). There were 63.47% of the proteins that included at least two peptides (Supplementary Figure 1B) and the most enriched protein masses are 20–30 and 30–40 kDa, followed by 40–50, 50–60, and 10–20 kDa proteins (Supplementary Figure 1C).

Compared with the control, 469 proteins showed significantly changed accumulation (244 with increased accumulation and 225 with decreased accumulation) in the leaves of Ca(NO_3_)_2_-treated seedlings (Figure 2B). Moreover, a total of 172 (79 with increased accumulation and 93 with decreased accumulation) and 442 (251 with increased accumulation and 191 with decreased accumulation) proteins with significantly different accumulation were identified in the BR/control and Ca+BR/Ca comparison groups, respectively (Figure 2B).

### Functional Annotation of Differentially Changed Proteins

Functional annotations of the 172, 469, and 442 DCPs belong to the BR/control, Ca/control, and Ca+BR/Ca groups, respectively, (Figure 2B; Supplementary Figure 2; and Supplementary Tables 4–7) showed that the DCPs in the Ca/control were annotated into 47 functional terms, including 11 molecular function terms, 16 cellular component terms, and 20 biological process terms (Supplementary Figure 2A). Additionally, 442 DEPs in the Ca+BR/Ca were annotated to 47 functional groups, including 18, 17, and 12 terms in biological process, cellular component, and molecular function, respectively (Supplementary Figure 2B). The proteins were selected based on different GO terms shown in Figure 2C.

The DCPs were then blasted KEGG genes to retrieve their KEGG ortholog (KOs) and were subsequently mapped to the pathways in KEGG (Supplementary Figure 3). In the Ca(NO_3_)_2_-treated seedlings, the KEGG pathways were enriched in carbohydrate metabolism including pyruvate metabolism, glycolysis, glyoxylate, and dicarboxylate metabolism, and carbon fixation in the photosynthetic organisms involved in energy metabolism (Supplementary Figure 3A). In the Ca+BR-treated seedlings, the KEGG pathways were enriched in photosynthesis, carbon metabolism, propionate metabolism, pyruvate metabolism, and glycolysis (Supplementary Figure 3C). These results collectively indicated that compared with Ca(NO_3_)_2_ stress, foliar spraying with BR largely affected carbohydrate and energy metabolism pathways.

### Validation of Isobaric Tags for Relative and Absolute Quantification Data for Selected Proteins by Parallel Reaction Monitoring

The iTRAQ data were subsequently confirmed using Skyline software. The detailed data of the 25 target peptide fragments are shown in Supplementary Table 8. The accumulation of several antioxidant-related proteins, such as P30264, Q6X1D0, K4ASJ5, and K4ASJ6, and the light-harvesting complex chlorophyll A-B binding protein (P27524) elevated in the leaves of the Ca(NO_3_)_2_-treated seedlings. As shown in Supplementary Tables 4, 6, 8, the iTRAQ validation showed that Ca(NO_3_)_2_ stress increased the accumulation of Chlorophyll a-b binding protein (P27524, P27489, K4CXU8, K4C768) and ferredoxin (K4D1V7) and decreased the accumulation of cytochrome P450-type monooxygenase 97A29 (D2CV80). These results were consistent with the iTRAQ results, which indicated that Ca(NO_3_)_2_ stress affected photosynthesis and antioxidant defense in the leaves of the tomato seedlings.

Foliar spraying with BR increased the accumulation of antioxidant-related proteins such as POD (K4ASJ6, K4ASJ5) and cysteine proteinase 3 (Q40143), the proteins involved in the KEGG pathways of biosynthesis of amino acids (sly01230), photosynthesis-antenna proteins (sly00196), and protein processing in the endoplasmic reticulum (sly04141; Supplementary Tables 4, 5, 8).

Exogenous BR also affected the photosynthesis-related proteins under Ca(NO_3_)_2_ stress. Compared with Ca(NO_3_)_2_ stress, the accumulation of chlorophyll a-b binding protein (K4DC08) down-regulated, while that of PSBR (Q40163), PsbQ 543931 (Q672Q6), psbH (A0A0C5CEE1), and ferredoxin-1 (Q43517) up-regulated in the Ca+BR treatment (Supplementary Tables 4, 7, 8). Moreover, the changes in protein accumulation detected by the PRM assay were consistent with the iTRAQ results, indicating that the iTRAQ results were sufficiently valid.

### Brassinolide Alleviated Ca(NO_3_)_2_-Induced Photosynthetic Inhibition

**FIGURE 3:**
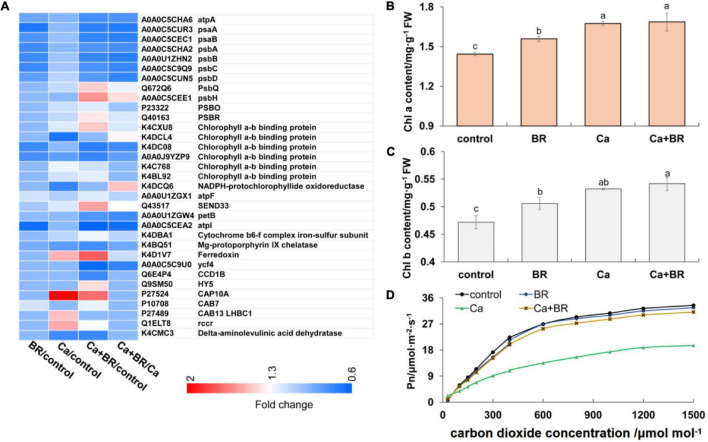
BR alleviated Ca(NO_3_)_2_-inhibited photosynthesis in tomato seedlings. **(A)** Heatmap of the differentially changed proteins involved in photosynthesis. **(B)** Chlorophyll a content. **(C)** Chlorophyll b content. **(D)** Photosynthesis-CO_2_ response curve. BR, 0.1 μmol/L BR; Ca, 100 mmol/L Ca(NO_3_)_2_; Ca+BR, 100 mmol/L Ca(NO_3_)_2_ stress plus foliar spraying with exogenous BR. Each data point represents the mean of three independent biological replicates (mean ± SD). Different letters above the bars indicate statistically significant differences (*P* < 0.05).

Proteomics analysis revealed that exogenous BR affected the accumulation of proteins involved in chlorophyll metabolism and photosynthesis in tomatoes under Ca(NO_3_)_2_ stress (Figure 3A). The key enzyme in the metabolism of porphyrin and chlorophyll is nicotinamide adenine dinucleotide phosphate (NADPH)-protochlorophyllide oxidoreductase (K4DCQ6). Calcium nitrate stress down-regulated the accumulation of K4DCQ6 in the tomato leaves. Moreover, in the pathway of porphyrin and chlorophyll metabolism, the significant increase of red chlorophyll catabolite reductase (Q1ELT8) under Ca(NO_3_)_2_ stress enhanced the electron transport in chlorophyll decomposition (Figure 3A). Foliar spraying with BR had no significant effect on the red chlorophyll catabolite reductase (Fragment; Q1ELT8) under salt stress, but K4DCQ6 was up-regulated in the “Ca+BR/Ca” group, and thereby modulating chlorophyll levels in the Ca+BR treatment (Figure 3A). Meanwhile, Ca(NO_3_)_2_ stress increased the chlorophyll contents in the tomato leaves; however, foliar spraying with BR did not affect the chlorophyll contents in the Ca(NO_3_)_2_-treated tomato seedlings (Figures 3B,C). Altogether, these results indicated that BR alleviated Ca(NO_3_)_2_ stress by improving the function and stability of the photosynthetic system in tomato leaves. Next, we evaluated the effects of BR and Ca(NO_3_)_2_ on the photosynthetic parameters. Foliar spraying with BR decreased the WUE and *Ls* in the tomato leaves (Figure 3D and Table 1). Calcium nitrate stress inhibited the *Pn* and *Tr*, and decreased the *Gs* and *Ci*, but increased the *Ls* and WUE in the tomato leaves (Figure 3D and Table 1). However, foliar spraying with BR improved the *Pn*, *Tr*, *Gs*, and *Ci*, but decreased the WUE and *Ls* in the Ca(NO_3_)_2_-treated seedlings (Figure 3D and Table 1).

**TABLE 1:** Effects of exogenous BR on the photosynthetic parameters in the leaves of tomato seedlings under Ca(NO_3_)_2_ stress.

	Pn	Ci	Gs	Tr	WUE	Ls
Control	17.11 ± 1.20b	265.39 ± 10.83b	0.15 ± 0.03c	2.39 ± 0.18b	7.11 ± 0.42b	0.44 ± 0.03b
BR	20.77 ± 0.31a	296.44 ± 4.79a	0.22 ± 0.01a	3.54 ± 0.28a	5.90 ± 0.42c	0.36 ± 0.01c
Ca	10.86 ± 0.69d	191.81 ± 12.41c	0.07 ± 0.01d	1.15 ± 0.26c	9.52 ± 1.76a	0.61 ± 0.02a
Ca+BR	14.51 ± 0.94c	255.68 ± 10.49b	0.17 ± 0.02b	3.30 ± 0.09a	4.54 ± 0.10d	0.39 ± 0.03c

*Error bars represent the SD (*n* = 3). Different letters indicate values that were significantly different at *P* < 0.05 according to Duncan’s test.*

### Brassinolide Alleviated Ca(NO_3_)_2_-Induced Oxidative Damage in Tomato Seedlings

**FIGURE 4:**
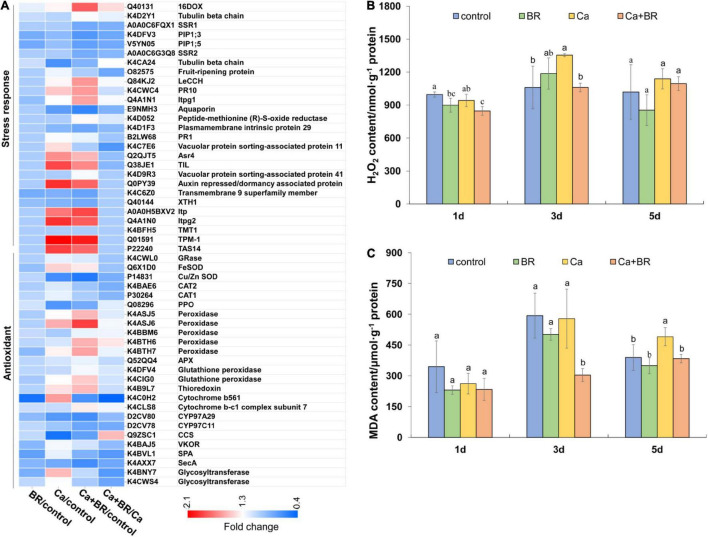
The degree of membrane lipid peroxidation in the leaves of tomato seedling subjected to Ca(NO_3_)_2_, foliar spraying with BR, or their combinations. **(A)** Heatmap of the differentially changed proteins involved in the antioxidant system. **(B)** Hydrogen peroxide (H_2_O_2_) content. **(C)** Malondialdehyde (MDA) content. BR, 0.1 μmol/L brassinolide; Ca, 100 mmol/L Ca(NO_3_)_2_; Ca+BR, 100 mmol/L Ca(NO_3_)_2_ stress plus foliar spraying with exogenous BR. Each data point represents the mean of three independent biological replicates (mean ± SD). Different letters above the bars indicate statistically significant differences (*P* < 0.05).

The overproduction of ROS and subsequent oxidative damage commonly occur under salt stress (Alexander et al., 2020; Yang et al., 2020). Proteomics analysis showed that Ca(NO_3_)_2_ stress affected the accumulation of proteins involved in stress responses and antioxidant defense, and exogenous BR increased the accumulation of antioxidant enzymes and proteins involved in the responses to osmotic stress in tomatoes under Ca(NO_3_)_2_ stress (Figure 4A). To further confirm the proteomics results, we investigated the levels of H_2_O_2_ and oxidative damage in the Ca(NO_3_)_2_-treated tomato plants. Calcium nitrate stress significantly increased the content of H_2_O_2_ and MDA at 3 and 5 days after the treatment, respectively. However, foliar spraying with BR significantly reduced the H_2_O_2_ accumulation and MDA content in the leaves of Ca(NO_3_)_2_-treated tomato seedlings after 3 and 5 days of treatment, respectively (Figures 4B,C).

**FIGURE 5:**
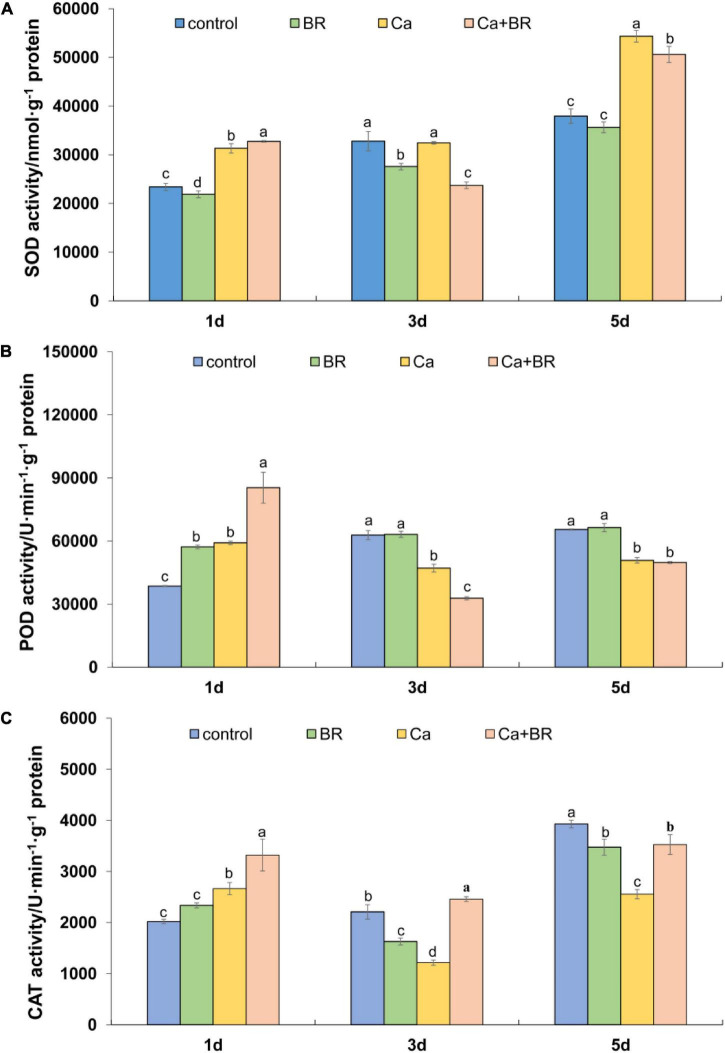
Antioxidant enzyme activity in the leaves of the tomato seedling subjected to Ca(NO_3_)_2_, foliar spraying with BR, or their combinations. **(A)** Superoxide dismutase (SOD) activity. **(B)** Peroxidase (POD) activity. **(C)** Catalase (CAT) activity. BR, 0.1 μmol/L brassinolide; Ca, 100 mmol/L Ca(NO_3_)_2_; Ca+BR, 100 mmol/L Ca(NO_3_)_2_ stress plus foliar spraying with exogenous BR. Each data point represents the mean of three independent biological replicates (mean ± SD). Different letters above the bars indicate statistically significant differences (*P* < 0.05).

The time-course of the antioxidant enzyme activity showed the differential effects of Ca(NO_3_)_2_ and BR. For instance, Ca(NO_3_)_2_ significantly increased the activity of SOD after 1 and 5 days, but not after 3 days of treatment. Unlike this trend, Ca(NO_3_)_2_ stress significantly increased the activity of POD and CAT 1 day after treatment but it reduced the activity of POD and CAT in the tomato leaves after 3 and 5 days compared with the control (Figures 5B,C). However, compared with the seedlings only exposed to Ca(NO_3_)_2_ stress, the activity of these three enzymes was significantly increased by BR treatment after 1 day of Ca(NO_3_)_2_ stress. Foliar spraying with BR reduced Ca(NO_3_)_2_-induced SOD activity (Figure 5A) and improved the CAT activity in the leaves of Ca(NO_3_)_2_-treated tomato seedlings after 3 and 5 days of treatment (Figure 5C). Meanwhile, foliar spraying with BR further reduced POD activity after 3 days in the Ca(NO_3_)_2_-treated tomato seedlings (Figure 5B). However, the activity of POD in the Ca+BR-treated tomato seedlings was not significantly different from that in the only Ca(NO_3_)_2_-treated tomato seedlings after 5 days of treatment.

**FIGURE 6:**
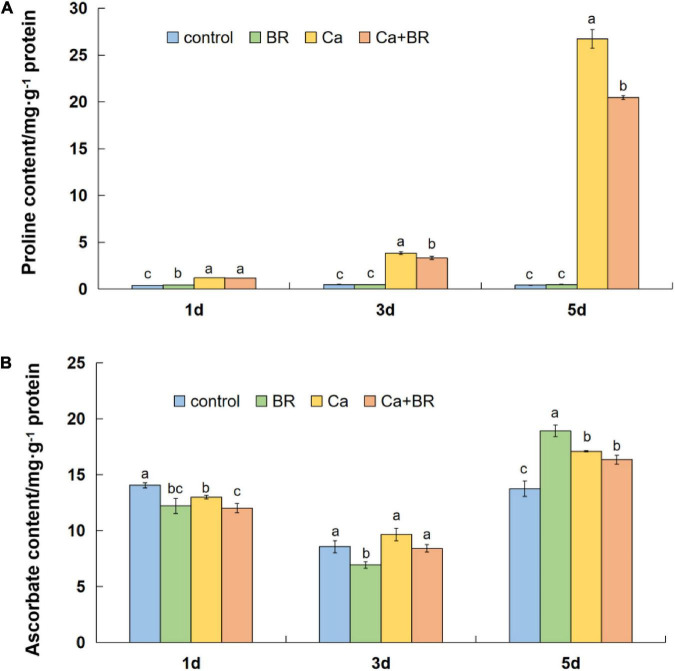
**(A)** Proline and **(B)** ascorbic acid content in the leaves of the tomato seedlings subjected to Ca(NO_3_)_2_, foliar spraying with BR, or their combinations. BR, 0.1 μmol/L brassinolide; Ca, 100 mmol/L Ca(NO_3_)_2_; Ca+BR, 100 mmol/L Ca(NO_3_)_2_ stress plus foliar spraying exogenous BR. Each data point represents the mean of three independent biological replicates (mean ± SD). Different letters above the bars indicate statistically significant differences (*P* < 0.05).

Calcium nitrate stress markedly increased the content of proline (Pro) in the tomato leaves throughout the experimental period. However, exogenous BR significantly reduced the Ca(NO_3_)_2_-induced increases in the Pro content after 3 and 5 days of Ca(NO_3_)_2_ stress, and the effect of BR was more profound with the prolongation of the treatment time (Figure 6A). Meanwhile, the AsA content differentially fluctuated with time in the Ca(NO_3_)_2_-treated tomato seedlings (Figures 6A,B). Compared with the control, the AsA content decreased after 1 day, however, it eventually increased after 5 days of Ca(NO_3_)_2_ stress (Figure 6B). Calcium nitrate alone or combined with BR did not alter the AsA accumulation at 3 days of the Ca(NO_3_)_2_ treatment. Also, the BR treatment on the Ca(NO_3_)_2_-treated tomato seedlings did not affect the AsA content compared with the only Ca(NO_3_)_2_-treated tomato seedlings after 3 and 5 days of the Ca(NO_3_)_2_ treatment. These results collectively supported the proteomics data and indicated that exogenous BR alleviated Ca(NO_3_)_2_-induced growth inhibition by increasing the antioxidant enzyme activity, thus, reducing the level of membrane lipid peroxidation, finally improving Ca(NO_3_)_2_ stress tolerance in tomatoes.

### Brassinolide Affected the Contents of Amino Acids in the Tomato Leaves Under Ca(NO_3_)_2_ Stress

**FIGURE 7:**
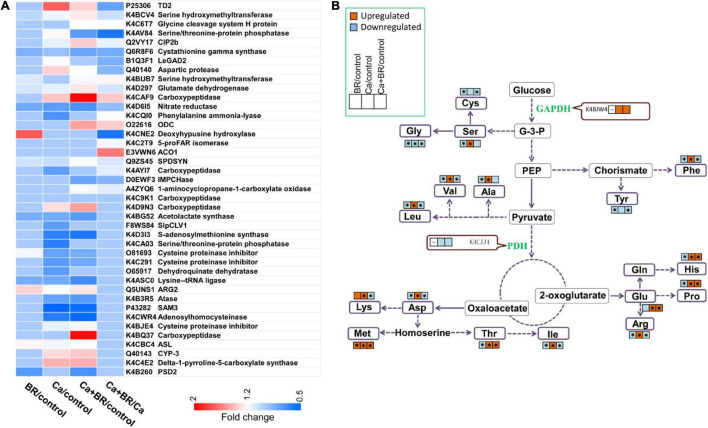
Changes in the content of amino acids and enzymes involved in the glycolysis and TCA cycle. **(A)** Heatmap of the differentially changed proteins involved in amino acid metabolism. **(B)** Amino acids or the enzymes that showed significantly higher or lower accumulation in the leaves of tomato seedlings compared with those in the untreated control seedlings are represented by red (up-regulated) and blue (down-regulated) boxes. Asterisks indicate statistically significant differences (*P* < 0.05).

**FIGURE 8:**
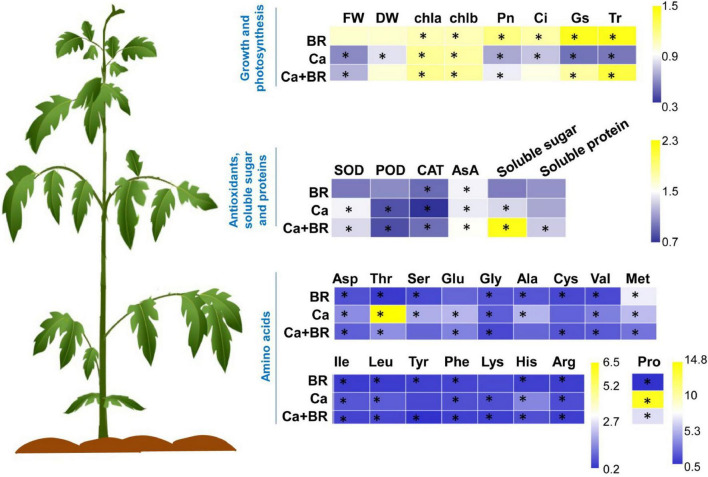
Brassinolide alleviated Ca(NO_3_)_2_-induced growth inhibition by modulating photosynthesis, antioxidants, osmoregulatory substances, and amino acid contents in tomato seedlings. The heatmap represents the fold change of each value compared with the corresponding untreated control. Asterisks indicate that values were significantly different compared with the untreated control plants at *P* < 0.05.

The proteomic analysis showed that Ca(NO_3_)_2_ stress disrupted the amino acid metabolism in the tomato leaves (Figure 7A). The phenylalanine (Phe) content increased under Ca(NO_3_)_2_ stress, while BR decreased the Phe content of the salt-stressed tomato seedlings. This change was negatively correlated with the decrease of PAL (K4CQI0) accumulation in the Ca/control and the increase of K4CQI0 accumulation in the Ca+BR/Ca (Figures 7, 8 and Supplementary Table 7). Moreover, the levels of amino acids such as threonine (Thr), glutamate (Glu), alanine (Ala), valine (Val), methionine (Met), isoleucine (Ile), leucine (Leu), histidine (His) and proline (Pro) increased in the Ca(NO_3_)_2_-treated tomato seedlings, thereby reprogramming the primary metabolism of plants to salt stresses, while after foliar spraying with BR, all the amino acid content decreased in the Ca(NO_3_)_2_-treated tomato seedlings (Figures 7, 8). All of the amino acids tested, except glycine (Gly), cysteine (Cys), and tyrosine (Tyr), showed markedly increased content in the leaves of the Ca(NO_3_)_2_-treated seedlings. Foliar spraying with BR decreased the contents of amino acids which were otherwise induced in the Ca(NO_3_)_2_ treatment (Figure 7B and Supplementary Table 9).

### Brassinolide Improved the Contents of Soluble Sugars and Soluble Proteins in Ca(NO_3_)_2_-Treated Seedlings

**FIGURE 9:**
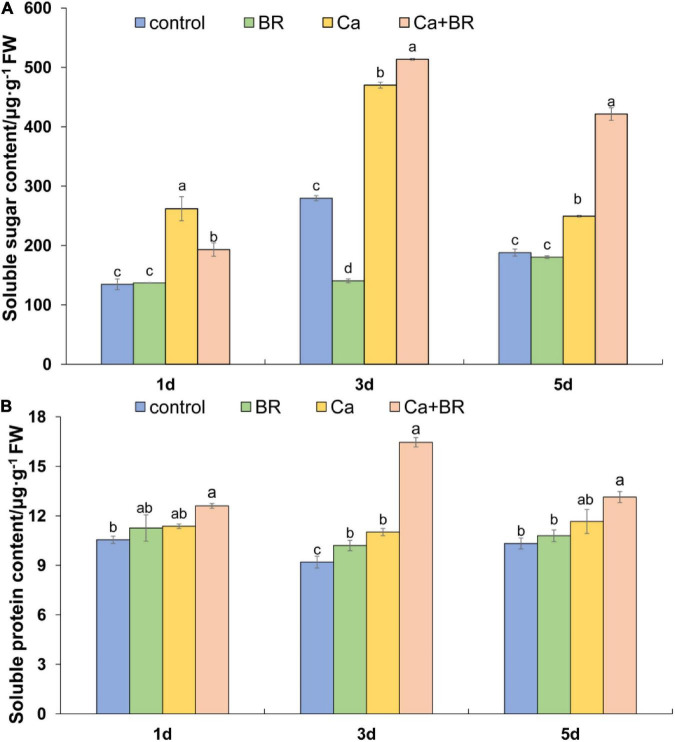
**(A)** Soluble sugar and **(B)** soluble protein content in the leaves of tomato seedlings subjected to Ca(NO_3_)_2_, foliar spraying with BR, or their combinations. BR, 0.1 μmol/L brassinolide; Ca, 100 mmol/L Ca(NO_3_)_2_; Ca+BR, 100 mmol/L Ca(NO_3_)_2_ stress plus foliar spraying with exogenous BR. Each data point represents the mean of three independent biological replicates (mean ± SD). Different letters above the bars indicate statistically significant differences (*P* < 0.05).

Soluble sugars and soluble proteins, as intracellular osmoregulatory substances, play important roles in modulating plant responses to salt stress. The KEGG pathway analysis involved in carbohydrate metabolism indicated that five pathways were containing 20 identified DCPs that were enriched in the Ca(NO_3_)_2_-treated plants (viz.) glycolysis/gluconeogenesis, glyoxylate and dicarboxylate metabolism, propanoate metabolism, pyruvate metabolism, citrate cycle (TCA cycle). The Ca(NO_3_)_2_ treatment also up-regulated the accumulation of malate dehydrogenase (K4B6N4, K4DCV3, K4CW40), thereby promoting the TCA cycle; in contrast, they down-regulated the accumulation of pyruvate dehydrogenase E1 component subunit beta (K4CJJ4) in the tomato seedlings (Supplementary Table 10). The proteomic analysis also showed that BR modulated the sugar and protein metabolism pathways in the Ca(NO_3_)_2_-treated tomato seedlings (Supplementary Table 10). We thus speculated if BR alleviated salt-induced stress by increasing the contents of osmoregulatory substances. To address this question, we measured the levels of soluble sugars and soluble proteins in the tomato seedlings. Calcium nitrate stress increased the content of soluble sugar throughout the study period. Although foliar spraying with BR decreased the soluble sugar content after 1 day of salt stress, BR further increased the content of leaf soluble sugars after 3 and 5 days of salt stress compared with the tomato seedlings only treated with Ca(NO_3_)_2_. The soluble protein content more or less increased in the Ca(NO_3_)_2_-treated tomato seedlings throughout the study period, whereas foliar spraying with BR increased the content of the soluble proteins significantly at 3 days after the Ca(NO_3_)_2_ treatment (Figures 9A,B). Notably, the positive regulatory effect of BR was more profound on the soluble sugars than soluble proteins under Ca(NO_3_)_2_ stress after 5 days.

## Discussion

Secondary salinization caused by Ca(NO_3_)_2_ deposition in greenhouse soil is a major problem in tomato cultivation (Zhang et al., 2020). In this study, we found that Ca(NO_3_)_2_ stress repressed photosynthesis and induced oxidative damage in tomato seedlings. However, foliar spraying with BR showed a protective effect on alleviating Ca(NO_3_)_2_ stress by modulating the antioxidant capacity, photosynthesis, energy supply, and carbon/nitrogen metabolism processes in tomato seedlings (Figure 8).

Salt stress triggers excessive H_2_O_2_ accumulation, which induces lipid peroxidation and subsequently, oxidative damage (Yang and Guo, 2017). Thus ROS removal is a key strategy to minimize salt-induced oxidative damage, which largely relies on the efficient function of the antioxidant system (Wang and Huang, 2019; Wang et al., 2019). Previous studies showed that BR application elevated the activity of antioxidant enzymes, including ascorbate peroxidase (APX) activity in tomatoes, which conferred tolerance to oxidative stress (Claussen, 2005; Guo et al., 2018). Consistent with this, we found that exogenous BR-induced the rapid elevation of the activity of antioxidant enzymes such as SOD, POD, and CAT repressed H_2_O_2_ burst 1 day after Ca(NO_3_)_2_ stress (Figure 5), and thereby exerting a protective effect of BR on plants in response to the initial phase of Ca(NO_3_)_2_ stress. Although a few inconsistencies were found between H_2_O_2_ accumulation and antioxidant enzyme activity upon BR treatment during the later phases, a significant decrease in the MDA content in the Ca+BR treatment indicated the alleviation of Ca(NO_3_)_2_-induced oxidative stress after 5 days of salt treatment. It is to be noted that H_2_O_2_ also plays a signaling role and it mediates BR-induced stress tolerance (El-Mashad and Mohamed, 2012). From that context, indifferent H_2_O_2_ accumulation between Ca and Ca+BR after 5 days of stress cannot be conducive to the inefficacy of BR treatment in minimizing ROS. In the study on water dropwort, it was found that stress conditions could promote the accumulation of Pro and soluble sugar in plants (Kumar et al., 2021). However, the effects of exogenous BR on Pro accumulation under stress were inconsistent in different studies. There are pieces of evidence that showed a positive correlation between the accumulation of Pro and stress tolerance in plants (Claussen, 2005), but the role of Pro in the osmotolerance of plants is also controversial (Nanjo et al., 1999). In our study, foliar spraying with BR reduced Pro accumulation in salt-stressed tomatoes, and we speculate that Pro might act as a stress indicator. Moreover, exogenous BR-induced the differential regulation of Pro, soluble sugar, and soluble protein contents from 1 to 5 days of Ca(NO_3_)_2_ stress, indicating the more complex role of BR action in the production of antioxidant and osmotic adjustment substances, which appears to be specific to the temporal context, but increased the soluble sugar content and decreased the MDA content after 5 days which signified the role of BR in protecting plants from long-term osmotic stress and oxidative damage.

Under abiotic stress, plants develop thicker leaves with a thicker palisade mesophyll and a higher ratio of palisade mesophyll to spongy mesophyll thickness accompanied by lower photosynthetic pigment content (Shi et al., 2014; Zhou et al., 2017). Moreover, adverse environmental factors often lead to disorganized leaf palisades and spongy mesophylls, thus causing reduced photosynthesis (Ivanova et al., 2009; Shi et al., 2014; Zhou et al., 2017, 2020; Wang et al., 2020). Consistent with this, Ca(NO_3_)_2_ stress disrupted the arrangement and structure of the epidermis, palisade mesophyll, and spongy mesophyll in the tomato leaves in the current study. On the contrary, BR plays a vital role in leaf morphogenesis (Zhang et al., 2019). Long-term dark treatment represses leaf primordia development in *Arabidopsis*, while BR supplementation induces leaf bud development in the seedlings under the long-term dark treatment (Nagata et al., 2000). In rice, *BR-deficient dwarf1* (*brd1*) mutants showed an obvious defect in the elongation of the stem and leaves (Hong et al., 2002). Consistent with these results, we found that exogenous BR improved the structure of the epidermis, palisade mesophyll, and spongy mesophyll in tomato leaves (Figure 1C). Moreover, BR has parallel links to cell-cycle progression (through S-phase Cyclin D-CDK and the anaphase-promoting complex) and cell-wall functions (through cell-wall extensibility or microtubule dynamics), which in turn affects the extension and morphology of the leaves (Kuluev et al., 2014). Mesophyll cells are the main sites of photosynthesis in plants. Neatly arranged and intact palisade mesophyll could promote light capture in plants (Jiang et al., 2011). The zigzag and continuous spongy mesophyll is conducive to optimal gas exchange, so its structural integrity ensures the normal operation of photosynthetic reaction. Exogenous BR application reduced the extent of damage of salt stress to the mesophyll structure, which might contribute to enhanced photosynthesis. However, the molecular mechanisms underlying the BR-mediated development of leaf primordium, leaf expansion, and leaf dorsiventral polarity establishment require further in-depth research.

Plants regulate the photosynthetic rate by controlling the opening and closing of the stomata, nonetheless, the stomata may behave differently in response to salt stress (Oh et al., 2019). Studies have shown that salt stress inhibits the photosynthetic process in leaves mainly *via* stomatal and non-stomatal limitations (Yang et al., 2010). Despite the increasing *Ls*, Ca(NO_3_)_2_ stress significantly decreased *Pn*, *Gs*, *Tr*, and *Ci* in the leaves of the tomato seedlings, indicating that the decrease in *Pn* under Ca(NO_3_)_2_ stress was probably caused by the stomatal restriction. On the other hand, spraying BR could reduce the *Ls* of Ca(NO_3_)_2_-stressed seedlings, suggesting that exogenous BR might decrease the stomatal restriction of tomato leaves under Ca(NO_3_)_2_ stress, and enhance their photosynthetic capacity. These results collectively indicated that the reduction in the photosynthetic efficiency of the Ca(NO_3_)_2_-treated seedlings is related to stomatal constraints.

Salt stress represses the plant photosystem (PS) I and II activity, and causes damage to the PS I components, chlorophyll A-B binding proteins, and light-harvesting complex of chlorophyll A-B binding proteins, and thereby inhibits photosynthesis (Shunichi and Norio, 2008). Several studies have indicated that BR plays an important role in the regulation of photosynthesis (Li X. et al., 2016). Foliar spraying with BR improves the photosynthetic characteristics, actual photochemical efficiency, and quantum efficiency of leaves under normal temperature and short-term low-temperature stress (Zhang et al., 2020). In addition, BR treatment under low-temperature stress enhances the activity of PS II and antioxidant enzymes and protects the photosynthetic membrane from oxidative damage in cucumber plants (Fariduddin et al., 2011). Ferredoxin is involved in transferring PS I electrons to nicotinamide adenine dinucleotide phosphate (NADP^+^), and generating a reducing force of NADPH, leading to the promotion of CO_2_ assimilation in the Calvin cycle (Jiang et al., 2017), thus improving photosynthesis. Foliar spraying with BR up-regulated the accumulation of ferredoxin-1 (Q43517) in the “Ca+BR/Ca” group, which enhanced the PS I electron transfer in the Ca(NO_3_)_2_-treated tomato leaves. However, several proteins (PsaA, PsaB, psbC, psbB, PsbA, and PsbD) involved in the stabilization of PS I and PS II were down-regulated in the “Ca+BR/Ca” group (Figure 3A). This result could be explained by the fact that the application of exogenous BR might prevent excessive electron transfer under Ca(NO_3_)_2_ stress, thereby providing a protective mechanism to the photosynthetic system.

The KEGG pathway analysis indicated that the Ca(NO_3_)_2_ treatment affected the TCA cycle and carbon metabolism process in tomato seedlings. The expression of glyceraldehyde-3-phosphate dehydrogenase (GAPDH), a key enzyme involved in glycolysis (Zhang et al., 2011), has a positive correlation with stress tolerance in plants (Pelah et al., 1997). A previous study found that BR treatment increased the abundance of proteins involved in sugar synthesis (such as sucrose synthase, sorbitol dehydrogenase, and IRX15-LIKE-like) in apple nursery trees. In addition, the contents of starch, sucrose, fructose, glucose, and total soluble sugar were increased in BR-treated leaves (Zheng et al., 2018), suggesting that BR affects sugar contents by modulating carbohydrate metabolism-related proteins. In this study, we found that Ca(NO_3_)_2_ stress up-regulated GAPDH (K4BJW4) in tomato leaves, while foliar spraying with BR further up-regulated the accumulation of GAPDH (K4BJW4; Supplementary Table 10). These results suggested that BR promoted carbon metabolism (including glycolysis and TCA cycle) in salt-stressed tomato seedlings, thereby improving salt tolerance in plants.

Plants mitigate the damage caused by stress by controlling the absorption, synthesis, and degradation of different amino acids (Li et al., 2019). We found that Ca(NO_3_)_2_ stress disrupted amino acid metabolism in tomato plants. Similarly, Martino et al. (2003) found that the contents of Glu, Gln, Asp, and Asn in spinach leaves decreased, while Ile, Leu, and Tyr increased after 43 days of sodium chloride (NaCl) treatment. Moreover, Wu et al. (2014) found that the contents of Tyr and Phe were negatively correlated, while the contents of Asp and Glu were positively correlated with the resistance of rice to saline-alkali stress. Our results further supported the hypothesis that free amino acids in plant organs are key indicators of plant tolerance to stress (Van et al., 2020). These results collectively indicated that exogenous BR application reprogrammed amino acid metabolism in the Ca(NO_3_)_2_-treated tomato seedlings. Taken together, the findings revealed that BR promoted sugar metabolism and amino acid metabolism in Ca(NO_3_)_2_-stressed tomato seedlings, thereby improving salt tolerance in tomato plants.

## Conclusion

In summary, our results showed that Ca(NO_3_)_2_ stress inhibited plant growth by inducing oxidative stress, and repressing photosynthesis and amino acid accumulation in tomato seedlings. Proteomics analyses further revealed that Ca(NO_3_)_2_ treatment modulated the accumulation of proteins involved in photosynthesis, stress response, and antioxidant defense. Foliar spraying with BR improved photosynthesis efficiency, sugar and amino acid metabolism, energy supply, and defense responses by increasing the accumulation of proteins involved in the photosynthesis, TCA cycle, and antioxidant defense of Ca(NO_3_)_2_-treated tomato seedlings. These physiological results combined with proteomics analyses provided a deep insight into Ca(NO_3_)_2_-mediated salt stress and the roles of BR in improving the stress tolerance of tomato plants.

## Data Availability Statement

The original contributions presented in the study are publicly available. This data can be found here: PeptideAtlas, with DataSet Identifier PASS01682 (www.peptideatlas.org/PASS/PASS01682).

## Author Contributions

YZ and YS designed and supervised the research. YZ, HC, YS, YL, and SL performed most experiments. BL and LB characterized the phenotypes. YZ, MK, JX, and YS analyzed the data and wrote the manuscript. All authors contributed to the article and approved the submitted version.

## Conflict of Interest

The authors declare that the research was conducted in the absence of any commercial or financial relationships that could be construed as a potential conflict of interest.

## Publisher’s Note

All claims expressed in this article are solely those of the authors and do not necessarily represent those of their affiliated organizations, or those of the publisher, the editors and the reviewers. Any product that may be evaluated in this article, or claim that may be made by its manufacturer, is not guaranteed or endorsed by the publisher.

## Footnotes

1
https://www.blast2go.com/

2
http://www.genome.jp/kaas-bin/kaas_main

## References

[B1] Ahammed G. J. Li X. Liu A. Chen S. (2020). Brassinosteroids in plant tolerance to abiotic stress. *J. Plant Growth Regul.* 39 1451–1464. 10.1007/s00344-020-10098-0

[B2] Ahmad P. Abd A. Alyemeni M. Leonard W. Pravej A. Renu B. (2018). Exogenous application of calcium to 24-epibrassinosteroid pre-treated tomato seedlings mitigates NaCl toxicity by modifying ascorbate-glutathione cycle and secondary metabolites. *Sci. Rep.* 8:13515. 10.1038/s41598-018-31917-1 30201952 PMC6131545

[B3] Alexander C. Denise A. Renato D. Gilmar D. Priscila L. Felisberto G. (2020). Different methods of silicon application attenuate salt stress in sorghum and sunflower by modifying the antioxidative defense mechanism. *Ecotox Environ. Safe* 203:110964. 10.1016/j.ecoenv.2020.110964 32678754

[B4] An Y. Zhou H. Zhong M. Sun J. Shu S. Shao Q. (2016). Root proteomics reveals cucumber 24-epibrassinolide responses under Ca(NO3)2 stress. *Plant Cell Rep.* 35 1081–1101. 10.1007/s00299-016-1940-z 26931454

[B5] Anzu M. Koji T. Shin-Ichiro I. Yasuomi T. Toshinori K. (2019). Brassinosteroid induces phosphorylation of the plasma membrane H+-ATPase during hypocotyl elongation in *Arabidopsis thaliana*. *Plant Cell Physiol.* 5 935–944. 10.1093/pcp/pcz005 30649552

[B6] Arif S. Tahir I. Syed S. Riyaz A. Nisar S. (2017). Efficacy of 24-epibrassinolide in improving the nitrogen metabolism and antioxidant system in chickpea cultivars under cadmium and/or NaCl stress. *Sci. Hortic Amster.* 225 48–55. 10.1016/j.scienta.2017.06.063

[B7] Bailly C. Benamar A. Corbineau F. Come D. (1996). Changes in malondialdehyde content and in superoxide dismutase, catalase and glutathione reductase activities in sunflower seeds as related to deterioration during accelerated aging. *Physiol. Plantarum.* 97 104–110. 10.1111/j.1399-3054.1996.tb00485.x

[B8] Bates L. Waldren R. Teare I. (1973). Rapid determination of free proline for water-stress studies. *Plant Soil.* 39 205–207. 10.1007/BF00018060

[B9] Bishop G. Yokota T. (2001). Plants steroid hormones, brassinosteroids: current highlights of molecular aspects on their synthesis/metabolism, transport, perception and response. *Plant Cell Physiol.* 42 114–120. 10.1093/pcp/pce018 11230564

[B10] Bradford M. (1976). A rapid and sensitive method for the quantitation of microgram quantities of protein utilizing the principle of protein-dye binding. *Anal. Biochem.* 72 248–254. 10.1016/0003-2697(76)90527-3 942051

[B11] Chi C. Li X. Fang P. Xia X. Shi K. Zhou Y. (2020). Brassinosteroids act as a positive regulator of NBR1-dependent selective autophagy in response to chilling stress in tomato. *J. Exp. Bot.* 71 1092–1106. 10.1093/jxb/erz466 31639824

[B12] Claussen W. (2005). Proline as a measure of stress in tomato plants. *Plant Sci.* 168 241–248. 10.1016/j.plantsci.2004.07.039

[B13] Du J. Guo S. Sun J. Shu S. (2018). Proteomic and physiological analyses reveal the role of exogenous spermidine on cucumber roots in response to Ca(NO3)2 stress. *Plant Mol. Biol.* 97 1–21. 10.1007/s11103-018-0721-1 29633167

[B14] El-Mashad A. A. A. Mohamed H. I. (2012). Brassinolide alleviates salt stress and increases antioxidant activity of cowpea plants (*Vigna sinensis*). *Protoplasma* 249 625–635. 10.1007/s00709-011-0300-7 21732069

[B15] Fan H. Ding L. Xu Y. Du C. (2017). Seed germination, seedling growth and antioxidant system responses in cucumber exposed to Ca(NO3)2. *Hortic Environ. Bio.* 58 548–559. 10.1007/s13580-017-0025-4

[B16] Fariduddin Q. Yusuf M. Chalkoo S. Hayat S. Ahmad A. (2011). 28-homobrassinolide improves growth and photosynthesis in *Cucumis sativus* L. through an enhanced antioxidant system in the presence of chilling stress. *Photosynthetica* 49 55–64. 10.1007/s11099-011-0022-2

[B17] Gong H. Zhu X. Chen K. Wang S. Zhang C. (2005). Silicon alleviates oxidative damage of wheat plants in pots under drought. *Plant Sci.* 169 313–321. 10.1016/j.plantsci.2005.02.023

[B18] Guo J. Zhou R. Ren X. Jia H. Hua L. Xu H. (2018). Effects of salicylic acid, epi-brassinolide and calcium on stress alleviation and cd accumulation in tomato plants. *Ecotox Environ. Safe.* 157 491–496. 10.1016/j.ecoenv.2018.04.010 29685680

[B19] Hao S. Cao H. Wang H. Pan X. (2019). The physiological responses of tomato to water stress and re-water in different growth periods. *Sci. Hortic.* 249 143–154. 10.1016/j.scienta.2019.01.045

[B20] Henry R. J. (2019). Innovations in plant genetics adapting agriculture to climate change. *Curr. Opin. Plant Biol.* 13 1–6. 10.1016/j.pbi.2019.11.004 31836470

[B21] Hong Z. Ueguchi-Tanaka M. Shimizu-Sato S. Inukai Y. Fujioka S. Shimada Y. (2002). Loss-of-function of a rice brassinosteroid biosynthetic enzyme, C-6 oxidase, prevents the organized arrangement and polar elongation of cells in the leaves and stem. *Plant J.* 32 495–508. 10.1046/j.1365-313x.2002.01438.x 12445121

[B22] Ivanova L. Ronzhina D. Ivanov L. Stroukova L. V. Peuke A. Rennenberg H. (2009). Chloroplast parameters differ in wild type and transgenic poplars overexpressing gsh1 in the cytosol. *Plant Biol.* 11 625–630. 10.1111/j.1438-8677.2008.00146.x 19538400

[B23] Jiang Q. Li X. Niu F. Sun X. Hu Z. Zhang H. (2017). iTRAQ-based quantitative proteomic analysis of wheat roots in response to salt stress. *Proteomics* 17 1–13. 10.1002/pmic.201600265 28191739

[B24] Jiang Y. Cheng F. Zhou Y. Xia X. Shi K. Yu J. (2011). Interactive effects of CO2 enrichment and brassinosteroid on CO2 assimilation and photosynthetic electron transport in *Cucumis sativus*. *Environ. Exp. Bot.* 75 98–106. 10.1016/j.envexpbot.2011.09.002

[B25] Kuluev B. Knyazev A. Nikonorov Y. Chemeris A. (2014). Role of the expansin genes NtEXPA1 and NtEXPA4 in the regulation of cell extension during tobacco leaf growth. *Russ. J. Genet.* 50 489–497. 10.1134/S1022795414040061 25715472

[B26] Kumar S. Li G. Yang J. Huang X. Ji Q. Liu Z. (2021). Effect of salt stress on growth, physiological parameters, and ionic concentration of water dropwort (*Oenanthe javanica*) cultivars. *Front. Plant Sci.* 12:660409. 10.3389/FPLS.2021.660409 34234795 PMC8256277

[B27] Li C. Li Y. Bai L. He C. Yu X. (2016). Dynamic expression of mi RNAs and their targets in the response to drought stress of grafted cucumber seedlings. *Hortic Plant J.* 2 41–49. 10.1016/j.hpj.2016.02.002

[B28] Li G. Wu Y. Liu G. Xiao X. Wang P. Tian G. (2017). Large-scale proteomics combined with transgenic experiments demonstrates an important role of jasmonic acid in potassium deficiency response in wheat and rice. *Mol. Cell. Proteom.* 16 1889–1905. 10.1074/mcp.RA117.000032 28821602 PMC5671998

[B29] Li L. Wang M. Sabin S. P. Li C. Megha M. P. Chen F. (2019). Effects of elevated CO2 on foliar soluble nutrients and functional components of tea, and population dynamics of tea aphid, Toxoptera aurantii. *Plant Physiol. Bioch.* 145 84–94. 10.1016/j.plaphy.2019.10.023 31675526

[B30] Li L. Xing W. Shao Q. Shu S. Sun J. Guo S. (2015). The effects of grafting on glycolysis and the tricarboxylic acid cycle in Ca(NO3)2-stressed cucumber seedlings with pumpkin as rootstock. *Acta Physiol. Plant.* 37 1–10. 10.1007/s11738-015-1978-5

[B31] Li M. Zhang K. Long R. Sun Y. Kang J. Zhang T. (2017). Itraq-based comparative proteomic analysis reveals tissue-specific and novel early-stage molecular mechanisms of salt stress response in carex rigescens. *Environ. Exp. Bot.* 143 99–114. 10.1016/j.envexpbot.2017.08.010

[B32] Li Q. He J. (2013). Mechanisms of signaling crosstalk between brassinosteroids and gibberellins. *Plant Signal Behav.* 8:e24686. 10.4161/psb.24686 23603943 PMC3909037

[B33] Li Q. Wang J. Xiong M. Wei K. Zhou P. Huang L. (2018). iTRAQ-based analysis of proteins co-regulated by brassinosteroids and gibberellins in rice embryos during seed germination. *Int. J. Mol. Sci.* 19:3460. 10.3390/ijms19113460 30400353 PMC6274883

[B34] Li S. Zhang Y. Yao Q. Bai L. Hou L. Shi Y. (2020). Effects of brassinolide on seedling growth and osmotic regulation characteristics of tomato under iso-osmotic salt stress. *J. JNWAFU* 48 130–136+145.

[B35] Li T. Yun Z. Wu Q. Zhang Z. Liu S. Shi X. (2013). Proteomic profiling of 24-epibrassinolide-induced chilling tolerance in harvested banana fruit. *J. Proteom.* 35 729–740. 10.1016/j.jprot.2018.05.011 29852298

[B36] Li X. Guo X. Zhou Y. Shi K. Zhou J. Yu J. (2016). Overexpression of a brassinosteroid biosynthetic gene dwarf enhances photosynthetic capacity through activation of calvin cycle enzymes in tomato. *BMC Plant Biol.* 16:33. 10.1186/s12870-016-0715-6 26822290 PMC4730719

[B37] Martino C. Delfine S. Pizzuto R. Loreto F. Fuggi A. (2003). Free amino acids and glycine betaine in leaf osmoregulation of spinach responding to increasing salt stress. *New Phytol.* 158 455–463. 10.1046/j.1469-8137.2003.00770.x 36056506

[B38] Nagata N. Min Y. Nakano T. Asami T. Yoshida S. (2000). Treatment of dark-grown *Arabidopsis thaliana* with a brassinosteroid-biosynthesis inhibitor, brassinazole, induces some characteristics of light-grown plants. *Planta* 211 781–790. 10.1007/s004250000351 11144262

[B39] Nanjo T. Kabayashi M. Yoshiba Y. Kakubari Y. Yamaguchi-Shinozaki K. Shinozaki K. (1999). Antisence suppression of proline degradation improves tolerance to freezing and salinity *Arabidopsis thaliana*. *Febs. Lett*. 461, 205–210. 10.1016/S0014-5793(99)01451-9 10567698

[B40] Nie S. Huang S. Wang S. Mao Y. Liu J. Ma R. (2019). Enhanced brassinosteroid signaling intensity via SlBRI1 overexpression negatively regulates drought resistance in a manner opposite of that via exogenous BR application in tomato. *Plant Physiol. Biochem.* 138 36–47. 30844693 10.1016/j.plaphy.2019.02.014

[B41] Niu S. Wang N. Irfan M. Xu J.-y. Zhang Y.-j. Cai G.-x. (2019). Effect of fermentation broth of endophytic fungi on physiological and biochemical characteristics of tomato seedling under calcium nitrate stress. *Iran. J. Sci. Technol. Trans. A Sci*. 43, 1427–1432. 10.1007/s40995-018-0640-7

[B42] Oh K. Mekapogu M. Ki S. (2019). Effect of salinity stress on photosynthesis and related physiological responses in carnation (*Dianthus caryophyllus*). *Hortic Environ. Biote.* 60 831–839. 10.1007/s13580-019-00189-7

[B43] Ohtsuki K. Kawabata M. Kokura H. Taguchi K. (2016). Simultaneous determination of S-methylmethionine, vitamin U and free amino acids in extracts of green tea with an HPLC-amino acid analyzer. *Biosci. Biotech. Bioch.* 51 2479–2484. 10.1080/00021369.1987.10868414

[B44] Pelah D. Shoseyov O. Altman A. Bartels D. (1997). Water-stress response in aspen (*Populus tremula*): differential accumulation of dehydrin, sucrose synthase, GAPDH homologues, and soluble sugars. *J. Plant Physiol.* 151 96–100. 10.1016/S0176-1617(97)80043-0

[B45] Peterson A. Russell J. Bailey D. Westphall M. Coon J. (2012). Parallel reaction monitoring for high resolution and high mass accuracy quantitative, targeted proteomics. *Mol. Cell Proteom.* 11 1475–1488. 10.1074/mcp.O112.020131 22865924 PMC3494192

[B46] Poonam B. Monika M. Vishwakarma A. Jyoti B. Yadav S. (2015). AtROS1 overexpression provides evidence for epigenetic regulation of genes encoding enzymes of flavonoid biosynthesis and antioxidant pathways during salt stress in transgenic tobacco. *J. Exp. Bot.* 66 5959–5969. 10.1093/jxb/erv304 26116024 PMC4566984

[B47] Quan W. Liu X. Wang H. Chan Z. (2015). Comparative physiological and transcriptional analyses of two contrasting drought tolerant alfalfa varieties. *Front. Plant Sci.* 6:1256. 10.3389/FPLS.2015.01256 26793226 PMC4709457

[B48] Shi G. Li S. Wang X. Liu C. (2014). Leaf responses to iron nutrition and low cadmium in peanut: anatomical properties in relation to gas exchange. *Plant Soil.* 375 99–111. 10.1007/s11104-013-1953-0

[B49] Shu S. Gao P. Li L. Yuan Y. Sun J. Guo S. (2016). Abscisic acid-induced H2O2 accumulation enhances antioxidant capacity in pumpkin-grafted cucumber leaves under Ca(NO3)2 stress. *Front. Plant Sci.* 7:1489. 10.3389/fpls.2016.01489 27746808 PMC5043297

[B50] Shunichi T. Norio M. (2008). How do environmental stresses accelerate photoinhibition? *Trends Plant Sci.* 13 178–182. 10.1016/j.tplants.2008.01.005 18328775

[B51] Soares C. Sousa A. Pinto A. Azenha M. Teixeira J. Azevedo R. (2016). Effect of 24-epibrassinolide on ROS content, antioxidant system, lipid peroxidation and Ni uptake in *Solanum nigrum* L. under Ni stress. *Environ. Exp. Bot*. 122, 115–125. 10.1016/j.envexpbot.2015.09.010

[B52] Su Q. Zheng X. Tian Y. Wang C. (2020). Exogenous brassinolide alleviates salt stress in malus hupehensis rehd. by regulating the transcription of NHX-type Na+(K+)/H+ antiporters. *Front. Plant Sci.* 11:38. 10.3389/fpls.2020.00038 32117377 PMC7016215

[B53] Tang W. Deng Z. Oses-Prieto J. A. Nagi S. Zhu S. Zhang X. (2008). Proteomic studies of brassinosteroid signal transduction using prefractionation and two-dimensional DIGE. *Mol. Cell. Proteom.* 7 728–738. 10.1074/mcp.M700358-MCP200 18182375 PMC2401332

[B54] Tanveer A. Yusuf M. Ahamad A. Bashir Z. Saeed T. Fariduddin Q. (2019). Proteomic and physiological assessment of stress sensitive and tolerant variety of tomato treated with brassinosteroids and hydrogen peroxide under low-temperature stress. *Food Chem.* 289 500–511. 10.1016/j.foodchem.2019.03.029 30955642

[B55] Tong H. Xiao Y. Liu D. Gao S. Liu L. Yin Y. (2014). Brassinosteroid regulates cell elongation by modulating gibberellin metabolism in rice. *Plant Cell.* 26 43–76. 10.1105/TPC.114.132092 25371548 PMC4277228

[B56] Van Z. Zhang Y. Testerink C. (2020). Salt tolerance mechanisms of plants. *Annu Rev. Plant Biol.* 71 403–433. 32167791 10.1146/annurev-arplant-050718-100005

[B57] Wang J. Huang R. (2019). Modulation of ethylene and ascorbic acid on reactive oxygen species scavenging in plant salt response. *Front. Plant Sci.* 10:319. 10.3389/fpls.2019.00319 30936887 PMC6431634

[B58] Wang N. Gao G. Wang Y. Wang D. Wang Z. Gu J. (2020). Coordinated responses of leaf and absorptive root traits under elevated CO2 concentration in temperate woody and herbaceous species. *Environ. Exp. Bot.* 179:104199. 10.1016/j.envexpbot.2020.104199

[B59] Wang Y. Cao J. Wang K. Xia X. Shi K. Zhou Y. (2019). BZR1 mediates brassinosteroid-induced autophagy and nitrogen starvation in tomato. *Plant Physiol.* 179 671–685. 10.1104/pp.18.01028 30482787 PMC6426427

[B60] Wiśniewski J. Zougman A. Nagaraj N. Mann M. (2009). Universal sample preparation method for proteome analysis. *Nat. Methods* 6 359–362. 10.1038/nmeth.1322 19377485

[B61] Wu Z. Yang C. Yang M. (2014). Photosynthesis, photosystem II efficiency, amino acid metabolism and ion distribution in rice (*Oryza sativa* L.) in response to alkaline stress. *Photosynthetica* 52 157–160. 10.1007/s11099-014-0002-4

[B62] Yan M. Xie D. Cao J. Xia X. Shi K. Zhou Y. (2020). Brassinosteroid-mediated reactive oxygen species are essential for tapetum degradation and pollen fertility in tomato. *Plant J.* 102 931–947. 10.1111/tpj.14672 31908046

[B63] Yang G. Komatsu S. (2004). Microarray and proteomic analysis of brassinosteroid-and gibberellin-regulated gene and protein expression in rice. *Genom Proteom Bioinf.* 2 77–83. 10.1016/S1672-0229(04)02013-3 PMC5172446 15629047

[B64] Yang Y. Guo Y. (2017). Elucidating the molecular mechanisms mediating plant salt-stress responses. *New Phytol.* 217 523–539. 10.1111/nph.14920 29205383

[B65] Yang Y. Liu Q. Wang G. Wang X. Guo J. (2010). Germination, osmotic adjustment, and antioxidant enzyme activities of gibberellin-pretreated picea asperata seeds under water stress. *New Forest.* 39 231–243. 10.1007/s11056-009-9167-2

[B66] Yang Y. Lu Z. Li J. Tang L. Jia S. Feng X. (2020). Effects of Ca(NO3)2 stress on mitochondria and nitrogen metabolism in roots of cucumber seedlings. *Agron* 10:167. 10.3390/agronomy10020167

[B67] Yuan L. Du J. Yuan Y. Shu S. Sun J. Guo S. (2014). Effects of 24-epibrassinolide on ascorbate-glutathione cycle and polyamine levels in cucumber roots under Ca(NO3)2 stress. *Acta Physiol. Plant.* 35 253–262. 10.1007/s11738-012-1071-2

[B68] Zhang F. Lu K. Gu Y. Zhang L. Li W. Li Z. (2019). Effects of low-temperature stress and brassinolide application on the photosynthesis and leaf structure of tung tree seedlings. *Front. Plant Sci.* 10:1767. 10.3389/fpls.2019.01767 32082338 PMC7005101

[B69] Zhang G. Liu Z. Zhou J. Zhu Y. (2008). Effects of Ca(NO3)2 stress on oxidative damage, antioxidant enzymes activities and polyamine contents in roots of grafted and non-grafted tomato plants. *Plant Growth Regul.* 56 7–19. 10.1007/s10725-008-9281-8

[B70] Zhang X. Rao X. Shi H. Li R. Lu Y. (2011). Overexpression of a cytosolic glyceraldehyde-3-phosphate dehydrogenase gene OsGAPC3 confers salt tolerance in rice. *Plant Cell Tiss Org.* 107 1–11. 10.1007/s11240-011-9950-6

[B71] Zhang Y. Li S. Liang Y. Zhao H. Hou L. Shi Y. (2018). Effects of exogenous spermidine and elevated CO2 on physiological and biochemical changes in tomato plants under iso-osmotic salt stress. *J. Plant Growth Regul.* 37 1222–1234. 10.1007/s00344-018-9856-1

[B72] Zhang Y. Yao Q. Shi Y. Li X. Hou L. Xing G. (2020). Elevated CO2 improves antioxidant capacity, ion homeostasis, and polyamine metabolism in tomato seedlings under Ca(NO3)2 -induced salt stress. *Sci. Hortic.* 273:109644. 10.1016/j.scienta.2020.109644

[B73] Zhen A. Zhang Z. Jin X. Liu T. Ren W. Hu X. (2018). Exogenous GABA application improves the NO3 -N absorption and assimilation in Ca(NO3)2-treated muskmelon seedlings. *Sci. Horticu Amster.* 227 117–123. 10.1016/j.scienta.2017.09.025

[B74] Zheng L. Ma J. Li Z. Gao C. Zhang D. Zhao C. (2018). Revealing critical mechanisms of BR-mediated apple nursery tree growth using iTRAQ-based proteomic analysis. *J. Proteom.* 173 139–154. 10.1016/j.jprot.2017.12.007 29277643

[B75] Zhong X. Wang Z. Xiao R. Wang Y. Xie Y. Zhou X. (2017). iTRAQ analysis of the tobacco leaf proteome reveals that RNA-directed DNA methylation (RdDM) has important roles in defense against geminivirus-betasatellite infection. *J. Proteom.* 152 88–101. 10.1016/j.jprot.2016.10.015 27989946

[B76] Zhou X. Wang Y. Shen S. (2020). Transcriptomic comparison reveals modifications in gene expression, photosynthesis, and cell wall in woody plant as responses to external pH changes. *Ecotox Environ. Safe.* 203:111007. 10.1016/j.ecoenv.2020.111007 32888586

[B77] Zhou Y. Huang L. Wei X. Zhou H. Chen X. (2017). Physiological, morphological, and anatomical changes in *Rhododendron agastum* in response to shading. *Plant Growth Regul.* 81 1–8. 10.1007/s10725-016-0181-z

[B78] Zhu L. Jia X. Li M. Wang Y. Zhang J. Hou J. (2021). Associative effectiveness of bio-organic fertilizer and soil conditioners derived from the fermentation of food waste applied to greenhouse saline soil in shan dong province, China. *Appl. Soil. Ecol.* 167:104006. 10.1016/J.APSOIL.2021.104006

