# Supplementary material for: Comparative Physiological and Proteomic Analyses Reveal the Mechanisms of Brassinolide-Mediated Tolerance to Calcium Nitrate Stress in Tomato
Source: Front Plant Sci. 2021 Nov 17;12:724288. doi: 10.3389/fpls.2021.724288 (PMC8636057; doi:10.3389/fpls.2021.724288)
Supplement: Supplementary file 1 [file Data_Sheet_1.DOCX]

**Supplemental files**

**Table S1-S10** See EXCEL files.

**Supplemental figures**


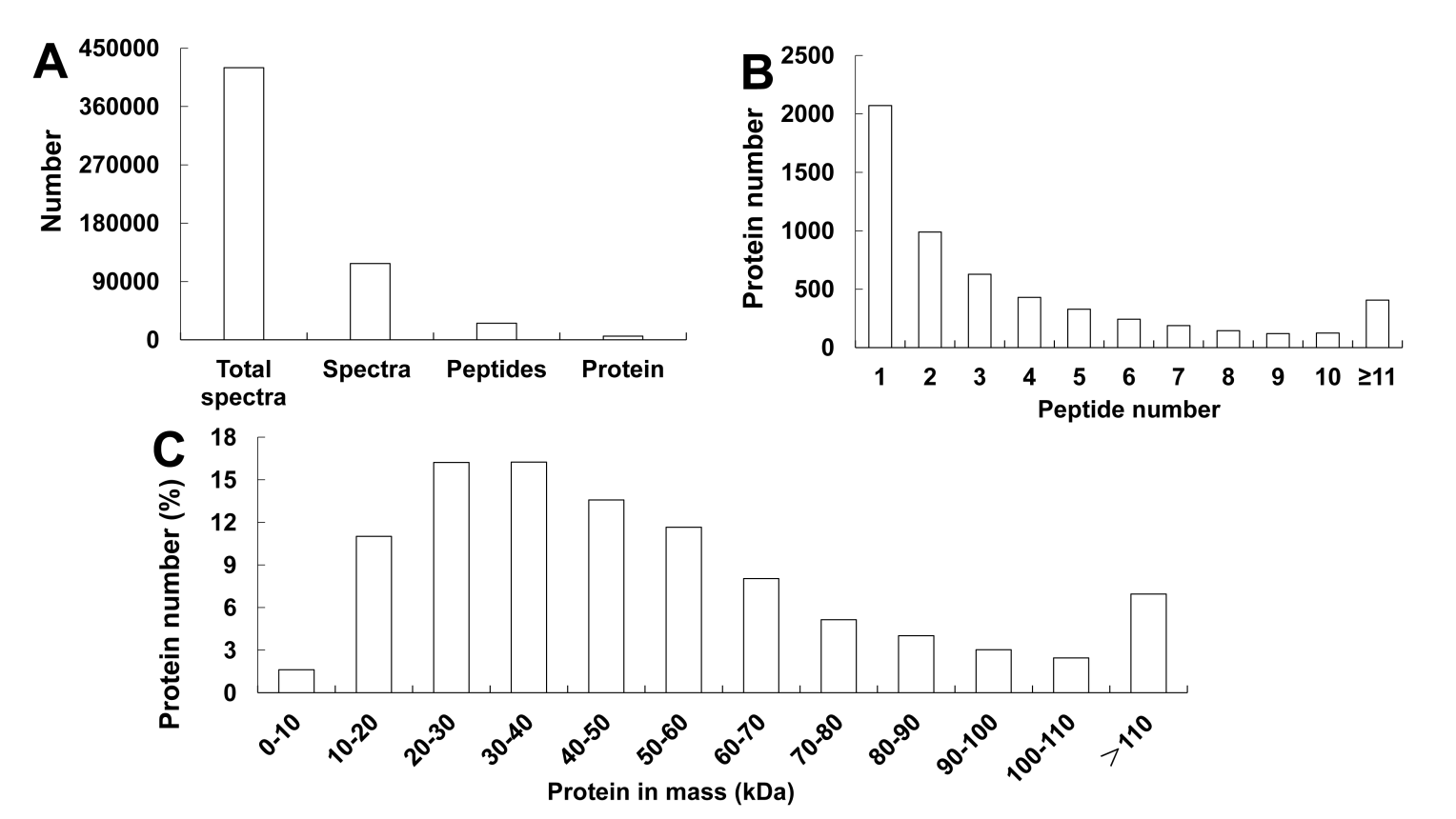


**Fig. S1 Mass detection results.**

**
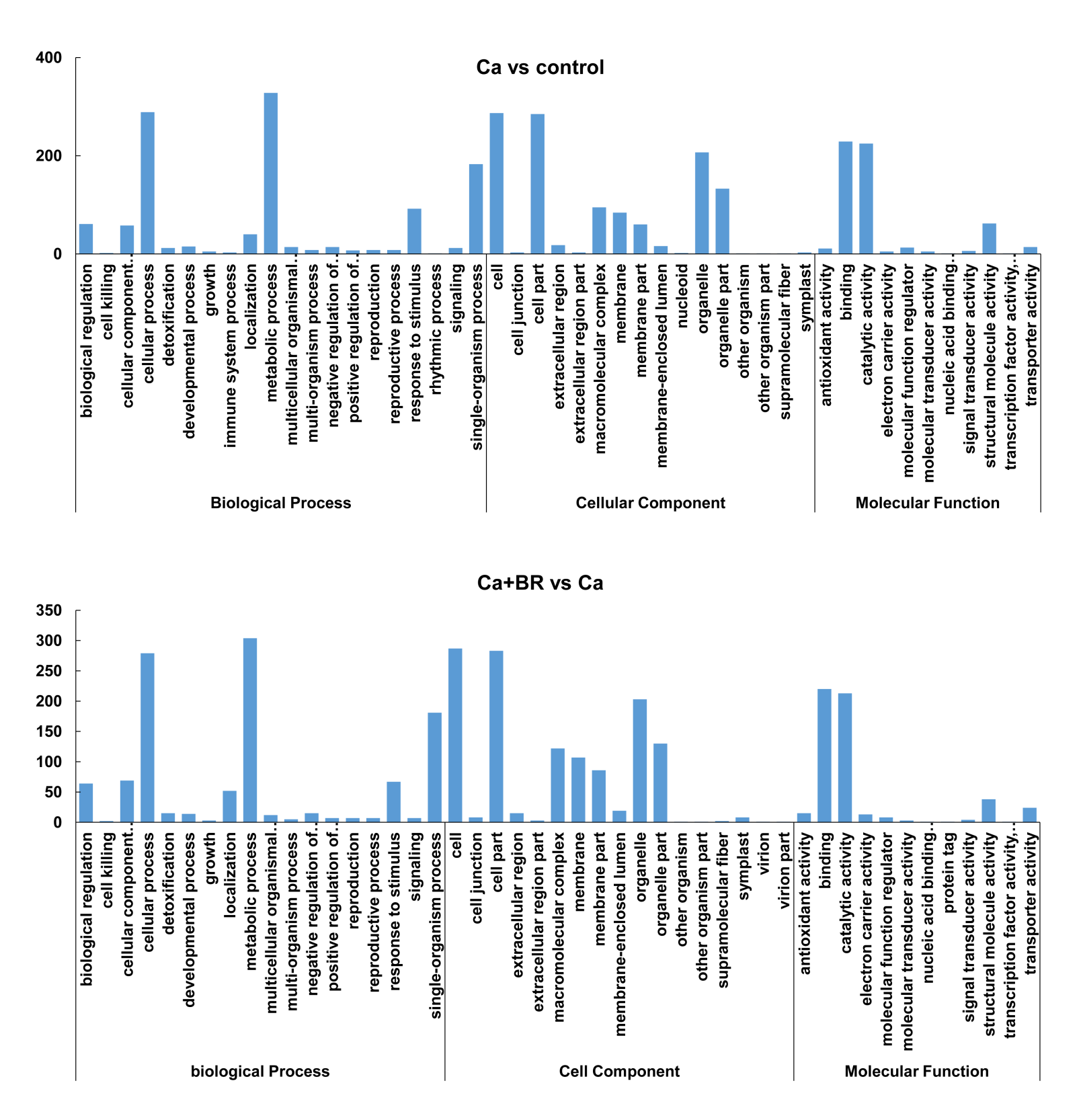
**

**Fig. S2 GO annotation of the differentially changed proteins based on their predicted molecular, including biological process, cellular component and molecular function.** BR, brassinolide; Ca, 100mmol/L Ca(NO_3_)_2_; Ca+BR, 100mmol/L Ca(NO_3_)_2_ stress plus foliar spraying exogenous BR.

**
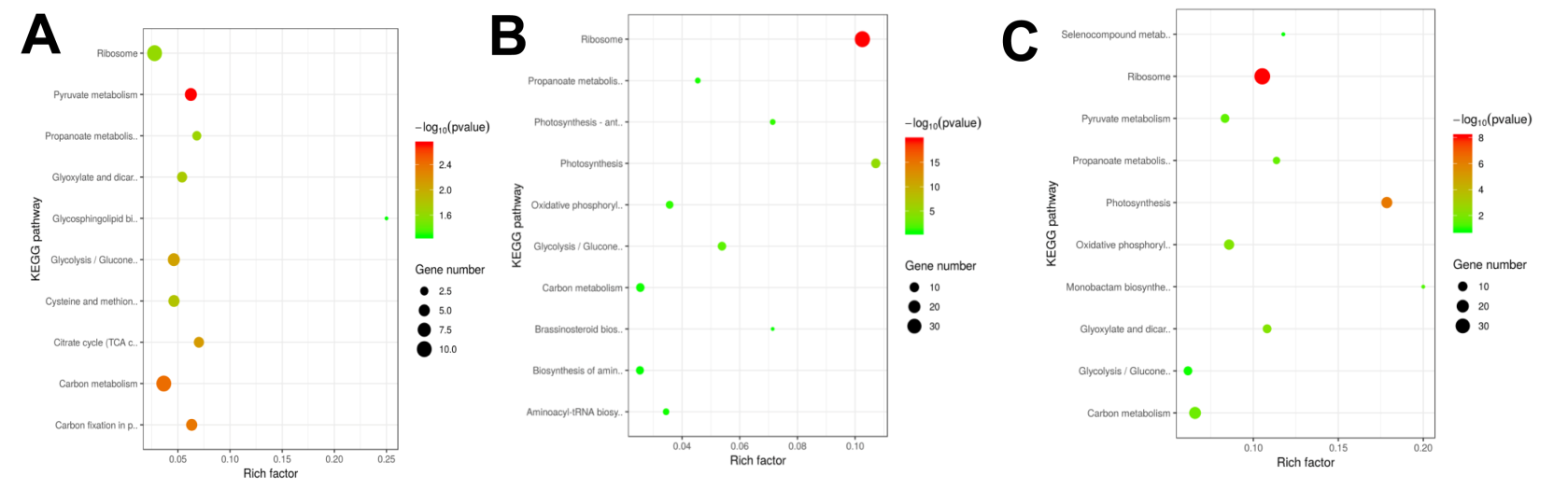
**

**Fig. S3 Kyoto Encyclopedia of Genes and Genomes pathway of the differentially changed proteins. A,** the TOP 10 pathways of unique DEPs induced by Ca(NO_3_)_2_ stress. **B**, the TOP 10 pathways of unique DEPs induced by BR under CK cultivation. **C**, the TOP 10 pathways of unique DEPs induced by BR under Ca(NO_3_)_2_ stress.

**Supplemental Methods**

**Protein extraction, digestion, iTRAQ labeling, and strong cation exchange (SCX) chromatography**

Second fully expanded leaves of the tomato seedlings were used for the extraction of total protein . Briefly,1.0 g of leaf sample from every biological replicate were finely crushed in liquid N_2_ and mixed with 10% (w/v) trichloroacetic acid/acetone solution having 65 mM dithiothreitol (DTT) for 1 h (-20°C). Afterwards, extracted sample was centrifuged for 45 min at 10,000×*g* and the obtained pellet was vacuum-dried and solubilized in 1/10 volumes of SDT buffer (4% SDS, 100 mM DTT, and 150 mM Tris-HCl, pH 8.0). After being incubated for 3 min, the suspended solution was ultrasonicated (80 w, 10 s ultrasonic at a time, every 15 s, and 10 times), and re-incubation at 100°C for 3 min followed by a centrifugation at 13,000×*g* at 25°C for 10 min. The protein content in each sample was calculated by BCA Protein Assay Reagent (Promega, USA) and sample were stored at -80°C until use.

Protein digestion was performed according to the FASP procedure described by Wisniewski^32^ and the resulting peptide mixture was marked according to the manufacturer’s instructions (AB SCIEX，Framingham，USA) with 8-plex isobaric tags for relative and absolute quantification (iTRAQ). Briefly, 200 μg of proteins were incorporated into 30 μl STD buffer (4% SDS, 100 mM DTT, 150 mM Tris-HCl pH 8.0). The detergent, DTT and other low-molecular-weight components were removed using UA buffer (8 M Urea, 150 mM Tris-HCl, pH 8.0) by repeated ultrafiltration (Microcon units, 30 kD). Then, 100 μl 0.05 M iodoacetamide in UA buffer was added to block reduced cysteine residues and the samples were incubated for 20 min in darkness. The filters were washed with 100 μl UA buffer for three times followed by addition of 100 μl DS buffer (50 mM triethylammonium bicarbonate at pH 8.5) twice. Finally, the protein suspensions were digested with 2 μg trypsin (Promega , Mannheim, Germany) in 40 μl DS buffer for overnight at 37 °C, and the resulting peptides were collected as a filtrate. The peptide content was estimated by UV light spectral density at 280 nm using an extinctions coefficient of 1.1 of 0.1% (g/l) solution and calculated on the basis of the frequency of tryptophan and tyrosine in vertebrate proteins.

Each iTRAQ reagent was dissolved in 70 μl of ethanol and added to the respective peptide mixture. The samples were multiplexed and vacuum dried. The iTRAQ labelled peptides were fractionated by SCX chromatography using the AKTA purifier system (GE Healthcare, Massachusetts, USA). The dried peptide mixture was reconstituted and acidified with 2 ml buffer A (10 mM KH_2_PO_4_ in 25% of ACN, pH 2.7) and loaded onto a PolySULFOETHYL 4.6×100 mm column (5 µm, 200 Å, PolyLC Inc, Maryland, USA). The peptides were eluted at a flow rate of 1 ml/min with a gradient of 0-10% buffer B (500 mM KCl, 10 mM KH_2_PO_4_ in 25% of ACN, pH 2.7) for 2 min, 10-20% buffer B for 25 min, 20-45% buffer B for 5 min, and 50-100% buffer B for 5 min. The elution was monitored by obtaining an absorbance at 214 nm, and fractions were collected every 1 min. The collected fractions (approximately 30 fractions) were finally combined into 10 pools and desalted on C18 Cartridges [Empore SPE Cartridges C18 (standard density), bed I.D. 7 mm, volume 3 ml, Sigma , Santa Clara, CA, USA]. Each fraction was concentrated by vacuum centrifugation and reconstituted in 40 µl of 0.1% (v/v) trifluoroacetic acid. All samples were stored at -80°C until LC-MS/MS analysis.

Liquid Chromatography (LC)-Electrospray Ionization (ESI) Tandem MS (MS/MS)

LC-MS/MS experiment was performed on a Q Exactive mass spectrometer coupled to Easy nLC (Proxeon Biosystems, now Thermo Fisher Scientific Waltham, MA, USA). A volume of 10 μl of each fraction was injected for LC-MS/MS analysis. The peptide mixture (5 μg) was loaded onto a the C18-reversed phase column (15 cm long, 75 μm inner diameter) packed in-house with RP-C18 5μm resin in buffer A (0.1% Formic acid) and separated with a linear gradient of buffer B (80% acetonitrile and 0.1% Formic acid) at a flow rate of 250 µl/min controlled by IntelliFlow technology over 140 min. The MS data was acquired using a data-dependent top 10 method, dynamically choosing the most abundant precursor ions from the survey scan (300–1800 m/z) for higher-energy collisional dissociation (HCD) fragmentation. Determination of the target value is based on predictive Automatic Gain Control (pAGC). Dynamic exclusion duration was set to 60 s. Survey scans were acquired at a resolution of 70,000 at m/z 200, and resolution for HCD spectra was set to 17,500 at m/z 200. Normalized collision energy was 30 eV and the underfill ratio, which specifies the minimum percentage of the target value likely to be reached at maximum fill time, was defined as 0.1%. The instrument was run with peptide recognition mode enabled. The LC-MS/MS analysis was carried out at HooGen Biotech, Shanghai, China.

**Sequence database searching and data analysis**

The MS/MS spectra were searched using Mascot search engine (Matrix Science, London, UK; version 2.2) embedded in Proteome Discoverer 1.3 (Thermo Electron, San Jose, CA) against the uniprot *Solanum lycopersicum* database (35921 sequences, download at 20180118) and the decoy database. The search parameters were as follows: Peptide mass tolerance: 20 ppm, MS/MS tolerance: 0.1 Da, Enzyme: Trypsin, max missed cleavage: 2, Fixed modification: Carbamidomethyl (C), iTRAQ8plex (K), iTRAQ8plex (N-term), Variable modification: Oxidation (M), FDR ≤ 0.01. Proteins were required to have at least two identified peptides. Data were normalized iteratively (across samples and spectra) according to the method reported previously^33^. Final protein abundances were calculated based on the average values of three biological replicates. Additionally, the fold change (FC) ratios of the identified proteins between two samples were used to assess the significance of any changes, while significant differences (*P* < 0.05) between the means of two samples were based on the Student’s t test. Differentially changed proteins were identified as proteins with FC ratio > 1.20 or < 0.83 (*P* < 0.05).

**Parallel reaction monitoring (PRM) analysis**

To further check the levels of protein abundance determined by iTRAQ analysis, additional quantification through liquid chromatography-parallel reaction monitoring (LC-PRM) MS analysis was applied. Briefly, the iTRAQ protocol was used for the preparation of 25 peptides. The stable isotope absolute quantification (AQUA) peptide was spiked in each sample and used as a standard internal reference. Tryptic peptides were loaded onto stage tips of C18 for desalting prior to reversed-phase chromatography on an Easy nLC-1200 system. The mobile phase consisted of solution A (0.1% formic acid) and solution B 0.1% formic acid acetonitrile aqueous solution (84% acetonitrile and 0.1% formic acid). The column was equilibrated with 95% solution A. Samples were injected into the Trap Column and passed through a Thermo scientific EASY column for gradient separation at a flow rate of 250 nl/min using the following linear gradient: 0-42 min, 5-23% solution B; 42-50 min, 23-40% solution B; 52-60min, the linear gradient of liquid B increased to 100% and maintained. After that, each sample was analyzed using a Q-Exactive HF (Thermo Scientific). Analysis time: 60 min. The mass spectrometer was performed in positive ion mode with the following parameters: AGC target value was set to 3e6, and the maximum ion injection time was 200 ms. Full MS scan from 300 to 1800 m/z was acquired with resolution of 60,000 (m/z 200), AGC target value was set to 3e6, and the maximum ion injection times was 200 ms. Full MS scans were followed by 25 PRM MS2 scans at 30,000 (m/z 200) resolution. The inclusion list was 25 entries and the isolation window was 1.6 m/z. The precursor ions were fragmented through HCD with normalized collision energy of 27 eV. The AGC target value was set to 3e6, and the maximum ion injection times were 120 ms. The analysis of raw data was realized via the Skyline 3.5.0 software (MacCoss Lab, University of Washington, USA)^34^, in which the intensity of the signal produced by a certain peptide sequence can be quantified according to each sample and referenced to standards via normalization of each protein.
